# Bulk mesophases modeling of ferroelectric nematic liquid crystals

**DOI:** 10.1038/s41467-026-78039-1

**Published:** 2026-10-03

**Authors:** Aditya Vats, Alenka Mertelj, Miha Ravnik

**Affiliations:** 1 Faculty of Mathematics and Physics, University of Ljubljana, Ljubljana, Slovenia; 2 Jožef Stefan Institute, Ljubljana, Slovenia

**Keywords:** Phase transitions and critical phenomena, Coarse-grained models, Liquid crystals

## Abstract

Ferroelectric nematic liquid crystals (FNLCs) are emerging materials that combine orientational order with spontaneous macroscopic polarization. They exhibit distinctive properties, such as polar domain formation, field-tunable textures, and strong electro-elastic couplings. However, due to the complexity of the underlying mechanisms, the bulk structure of the emergent mesophases is not yet fully understood, and a generalized description of all observed phases is still lacking. Here, we demonstrate the bulk mesophases of FNLCs as stabilized by a combination of flexoelectricity, elasticity, bulk ordering, and polarization-nematic couplings. Specifically, we develop Landau de-Gennes-like free energy framework that predicts and explains the structure of major experimentally observed ferroelectric nematic phases, including ferroelectric nematic, antiferroelectric splay nematic, apolar nematic, and isotropic phase, notably together with the correct phase transition sequences. Additionally, we predict the possible existence of new bend-instability-driven mesophase characterized by periodic splay-bend structure. Finally, we calculate a complete phase diagram of bulk ferroelectric nematics as dependent on the key coupling mechanisms, which could possibly enable the predictive design and applications of these novel emergent ferroelectric nematic materials.

## Abstract (web summary)

Ferroelectric nematic liquid crystals are an emerging class of materials in which nematic orientational order coexists with spontaneous macroscopic polarization. Vats et al. develop a free-energy framework to rationalize their bulk structures and predict potential bend-instability-driven mesophases.

## Introduction

Ferroelectric nematic liquid crystals (FNLCs) represent a phase that combines the long-range orientational order of nematics with spontaneous polarization. This polar order breaks inversion and mirror symmetries, distinguishing them from conventional nematic phases^1–4^. The concept of such a phase was first proposed by Max Born in 1916^5^. However, experimental realization was achieved only recently, with molecules such as RM734 and DIO exhibiting ferroelectric nematic behavior^2,6–9^. These systems rely sensitively on molecular design, particularly the presence of asymmetric terminal groups and lateral dipolar components. Even small chemical modifications (e.g., replacing *NO*_2_ with *CN*) can suppress ferroelectricity despite similar dipole moments, highlighting the role of dipole orientation and intermolecular packing^2,4,7^. FNLCs exhibit a macroscopic electric polarization coupled to the nematic director, leading to fundamentally new electro-optic responses and topological behaviors. This unique coupling opens the door to novel functionalities in display technology, tunable photonics, and low-energy memory devices^1,2,10–14^. From a fundamental point of view, FNLCs open new avenues for studying spontaneous symmetry breaking, topological defects, and electro-mechanical couplings in soft matter. Moreover, the fluid nature of the nematic phase coexisting with long-range polar order challenges conventional classifications of ordered media. Unlike solid-state ferroelectrics, FNLCs offer reconfigurability and flow, allowing for dynamic control of polar textures and domain structures.

The primary quest following the creation of stable FNLCs has been to understand the consequences of coupling between the nematic and polar components on their equilibrium and dynamic behavior. Some intriguing experimental observations have been made in this direction^1,2,15–21^. For example, Sebastian et al. demonstrated that the transition from the nematic to the ferroelectric phase is a ferroelectric-ferroelastic phase transition in which the growth of the ferroelectric order is accompanied by the softening of the splay elastic constant^22^. Furthermore, they observed polar domains with sharply defined walls or Ising walls, indicating spontaneous symmetry breaking at macroscopic scales^23^. In another contribution, Chen et al. reported a spontaneous and reorientable polarization locally parallel to the director in the RM734 system^8^. Additionally, there have been reports of rich phase behavior in these materials, including ferroelectric smectic A, ferroelectric twist-bend, smectic C etc^12,19,24–28^. These findings point to a strong coupling between polarization and elastic degrees of freedom, leading to rich static and dynamic phenomena not observed in conventional nematics. The presence of sharp domain walls and field-responsive polar textures suggests an underlying interplay between topology, elasticity, and electrostatics.

Experimental realizations of ferroelectric nematic materials, notably in RM734 and its derivatives, have also prompted extensive theoretical efforts to understand their complex phases and dynamic behavior. Both mesoscopic continuum approaches and lattice-based simulations have been employed in an effort to map the experimentally observed phase behavior and capture the emergence of polar domains with correct domain walls and topological defects^29–37^. These modeling efforts have helped identify key factors, such as elastic anisotropy, flexoelectric coupling, and external fields, that influence pattern formation and phase stability. However, the underlying mechanisms that govern the full range of equilibrium structures and defect-mediated processes in FNLCs remain only partially understood, particularly in regimes where electrostatic and flexoelectric couplings are non-negligible. In these regimes, long-range interactions and non-linear feedback between polarization and director distortions can lead to emergent behaviors. Existing modeling efforts have been largely focused on identifying individual phases and thus lack generality. A theoretical formulation with the correct phases and their sequences is still missing. Developing predictive models that incorporate the necessary couplings remains a central goal in advancing both fundamental understanding and the technological applications of ferroelectric nematics.

In this work, we develop a comprehensive understanding of the bulk ferroelectric nematic phases. A Landau-de Gennes-like free energy framework is developed that incorporates flexoelectric interactions, polarization gradient elasticity, and anisotropic coupling within a unified theoretical framework, enabling systematic exploration of both static and dynamic FNLC phenomena. Through extensive numerical simulations, we successfully reproduce the rich bulk mesophases observed experimentally as well as their phase sequence. Notably, we also demonstrate the emergent defect structures, including defect walls, and also predict the existence of a novel splay-bend phase in the regime of weakly (orientationally) coupled polarization and director. Our approach directly reveals how flexo-polar couplings stabilize different phases with exotic textures and domain structures and provides a controlled setting to examine the interplay of elastic, flexoelectric, polar, and topological degrees of freedom. This makes it possible to isolate the contributions of specific couplings and to test how they influence phase stability or defect interactions. The insights gained from our approach not only complement recent experimental observations but indicate a possible way for predictive control of structure in ferroelectric nematics. Finally, the approach can serve as a robust tool for the exploration of these emerging materials.

## Results

### Free energy

Mesoscopic models have proven to be highly successful in reproducing large-scale physical behavior and reconciling theoretical predictions with experimental data in various condensed matter systems^38–40^. In complex fluids, a general and powerful framework for describing materials and their phase transitions is the Landau-de Gennes (LdG)-like free energy-based approach^41–44^. In the case of FNLCs, the orientational order of the constituent anisotropic molecules is described by two order parameters. A symmetric traceless **Q**-tensor for the nematic field and the polarization vector **P** that accounts for the spontaneous polar order inherent to the ferroelectric phase. The average orientation direction -the director **n**- of the ferroelectric nematic corresponds to the eigenvector of **Q** with the largest eigenvalue. Note that polarization **P** is typically aligned along the director; therefore, today multiple studies commonly use only a single order parameter (typically **n** or **P**)^9,15,18,45–47^. However, using two order parameters carries profound additional physical meaning. It allows one to independently capture nematic and polar symmetries, their mutual coupling, and situations in which the polarization and nematic order partially decouple, which we show is needed, for example, to correctly introduce the effects of anisotropic flexoelectric response of ferroelectric nematics. Furthermore, each field carries its own elastic, electrostatic and field-coupling energetics that lead to distinct responses, particularly for strong external fields, near interfaces, or under strong distortions.

The total free-energy density *f* is given in Eq. (1) and combines expansion for nematic order, elastic terms for **Q** distortions, polarization free energy with gradients, and **Q**-**P** flexoelectric coupling. Selected experimental observations^2,7,9,15,18,22,23^ play a crucial role in the way we construct the free energy and the coupling between the two-order parameters; specifically: (i) the nematic director and the polarization vector in current ferroelectric nematic materials are always nearly co-aligned^1,9^; (ii) the ferroelectric nematic materials energetically favor elastic splay deformations over bend^2,9^; and (iii) domain walls in the polarization field exhibit Ising-like character^9,22,23,48^. Taking these experimental observations as general assumptions, we write the bulk free energy *f* of ferroelectric nematic as:

1 $$f=	 \frac{A}{4}\,{{\mbox{Tr}}}\,({{{\bf{Q}}}}^{2})+\frac{B}{3}\,{{\mbox{Tr}}}\,({{{\bf{Q}}}}^{3})+\frac{C}{16}\,{{\mbox{Tr}}}\,{({{{\bf{Q}}}}^{2})}^{2}+\frac{L}{2}{\left\vert \nabla {{\bf{Q}}}\right\vert }^{2}\\ 	 \frac{\mu }{2}{\left\vert {{\bf{P}}}\right\vert }^{2}+\frac{\nu }{4}{\left\vert {{\bf{P}}}\right\vert }^{4}+\frac{\kappa }{2}{\left\vert \nabla {{\bf{P}}}\right\vert }^{2} -{\lambda }_{1}{{\bf{P}}}\cdot (\nabla \cdot {{\bf{Q}}})-{\lambda }_{5}{{\bf{P}}}\cdot \left[({{\bf{Q}}}\cdot \nabla )\cdot {{\bf{Q}}}\right]\\ 	-{\lambda }_{6}{{\bf{P}}}\cdot \left[{{\bf{Q}}}\cdot (\nabla \cdot {{\bf{Q}}})\right]-\eta \,({{\bf{P}}}\cdot {{\bf{Q}}}\cdot {{\bf{P}}}).$$

The first line contains the bulk Landau-de Gennes expansion for the nematic field, with coefficients *A*, *B*, and *C* governing the isotropic-nematic transitions and the overall equilibrium nematic degree of order *S*. The elastic contributions involve gradients of the **Q**-tensor, which penalize local spatial variations in the order parameter. We use one elastic constant approximation. One could naturally expand the free energy also to multiple elastic constants (e.g., *L*_2_, *L*_3_ terms), but we show this is not needed to reproduce all the main ferroelectric nematic phases. The second line accounts for the Landau-Ginzburg free energy for the polarization field. The parameter with *μ* = *μ*_0_(*T* − *T*_C_) effectively controls the temperature. *T* is the quench temperature, and *T*_C_ is the effective transition temperature for the polar-to-apolar transition. *μ*_0_ and *ν* control the onset and magnitude of the spontaneous polarization. *κ* describes spatial polarization stiffness and penalizes the local variation in the polarization field. The third line represents the key couplings between two order parameter fields. The term proportional to *λ*_1_ in the free energy is a linear flexoelectric coupling between **P** and the gradients of **Q**. When expressed in terms of the director field **n**, this term reduces to

2 $${\lambda }_{1}S\,{{\bf{P}}}\cdot \left[(\nabla \cdot {{\bf{n}}}){{\bf{n}}}+({{\bf{n}}}\cdot \nabla ){{\bf{n}}}\right],$$

which corresponds to a coupling between the polarization and the splay and bend deformations of the director field. Notably, at this leading order, the splay and bend enter symmetrically into the flexoelectric free energy, in essence equally favorable, generating polarization along the splay and bend direction. However, for today’s ferroelectric nematic materials (like RM734, DIO and their derivatives), this turns out to be an improper assumption (although used widely in literature), since at the molecular level, the electric dipoles are aligned to a very strong degree (typically, within ~ ±15^∘^) with the long molecular axis, which in turn implies that the splay can generate flexoelectric polarization but not bend. Therefore, to break this degeneracy and allow independent control of the flexoelectric response to splay and bend distortions, higher-order flexoelectric terms are introduced with coefficients *λ*_5_ and *λ*_6_. The combined effect of *λ*_1_, *λ*_5_, and *λ*_6_ terms can be recast into an effective form as:

3 $$	{e}_{1} {{\bf{P}}}\cdot (\nabla \cdot {{\bf{n}}}){{\bf{n}}}+{e}_{3}{{\bf{P}}}\cdot ({{\bf{n}}}\cdot \nabla ){{\bf{n}}}=-{\lambda }_{1}{{\bf{P}}}\cdot (\nabla \cdot {{\bf{Q}}})-{\lambda }_{5} \\ 	 {{\bf{P}}}\cdot \left[({{\bf{Q}}}\cdot \nabla )\cdot {{\bf{Q}}}\right]-{\lambda }_{6}{{\bf{P}}}\cdot \left[{{\bf{Q}}}\cdot (\nabla \cdot {{\bf{Q}}})\right],$$

where *e*_1_ and *e*_3_ represent the more common flexoelectric coefficients for splay and bend, respectively^49,50^. These coefficients can be explicitly expressed for uniaxial order in terms of the coupling parameters and scalar order parameter *S* as:

4 $${e}_{1}={\lambda }_{1}S-\frac{1}{d}{\lambda }_{5}{S}^{2}+\frac{(d-1)}{d}{\lambda }_{6}{S}^{2},$$

5 $${e}_{3}={\lambda }_{1}S+\frac{(d-1)}{d}{\lambda }_{5}{S}^{2}-\frac{1}{d}{\lambda }_{6}{S}^{2}.$$

Finally, by tuning *λ*_5_ and *λ*_6_, one can selectively enhance or suppress the coupling to splay or bend, allowing control over the resulting polar textures and deformation modes, as emergent in FNLCs.

The final term that we include in the total free energy is (with coefficient *η*) the standard dielectric coupling, given as the dyadic product of **Q** and **P**, as it is the term of the lowest order that respects the up-down symmetry and rotational invariance of the system. It is easy to see that this coupling term in terms of the director field corresponds to ~(**n **⋅ **P**)^2^, which, with the correct *η* sign, co-aligns two fields. The dielectric term, together with flexoelectric couplings, links orientational distortions of the director field to the polarization and energetically favors splay/bend configurations with nonzero polarization. While similar phases can, in principle, be obtained using anisotropic elastic constants and dielectric coupling, flexoelectric terms are fundamental to capture correct defect structures (i.e., Ising-like defect wall) and the corresponding response to external electric fields.

The free energy in Eq. 1 has multiple coefficients, which can be linked to different experimentally measured quantities. For example, *A*, *B* and *C* may be determined from the birefringence, the isotropic-nematic transition temperature, and the latent heat from DSC^49^. Elastic constant *L* is related to the Frank constants, accessible through Frederiks-threshold measurements^49,51^. *μ* and *ν* can be inferred from the spontaneous polarization **P**(*T*). Similarly, the polar stiffness *κ* could be determined from the width of polar domain walls in SHG microscopy^2,8,22^. The anisotropic coupling *η* can be accessed through the equilibrium angle between P and the director, accessible by SHG interferometry. The flexoelectric coupling coefficients *λ*_*i*_ are constrained through their effect on the splay and bend elastic constants. For example, integrating out **P** in the paraelectric regime suggests effective constants ${K}_{1}^{\,{\mbox{eff}}\,}=2L{S}^{2}-{e}_{1}^{2}/{\mu }_{\parallel }$ and ${K}_{3}^{\,{\mbox{eff}}\,}=2L{S}^{2}-{e}_{3}^{2}/{\mu }_{\perp }$ in our formulation. Here *e*_1_ and *e*_3_ are linear combinations of the *λ*_*i*_ (see Eq. (4)) and ${\mu }_{\parallel }=\mu -2\eta S\left(1-\frac{1}{d}\right)$ and ${\mu }_{\perp }=\mu+\frac{2\eta S}{d}$. These effective elastic constants are measured directly in the experiments^9,22,52^. We believe such a joint set of measurements, similar to those in other non-ferroelectric liquid crystal materials, can provide good constraints on the different FNLC material parameter values.

This free energy captures the essential mechanisms required to model bulk ferroelectric nematic liquid crystals, where the interplay between orientational elasticity, polarization, and their mutual coupling gives rise to rich phenomenology, including domain formation, defect structures, and field-responsive textures. To compare it with alternative approaches in the literature, one approach is director-based models, which represent orientation order through the implementation of a unit-vector director field **n** supplemented by polarization degrees of freedom **P** ^9,32–34,36,48,53^. Complementarily, FNLCs have also been modeled using simplified electrostatic interactions of polar rods, capturing the role of density and molecular charge distribution in stabilizing polar order^54^. While such approaches successfully capture multiple qualitative characteristics of ferroelectric nematic phases, they knowingly exhibit two fundamental limitations: (i) the director formalism is inherently incapable of describing variations in the degree of orientational ordering (i.e., scalar order parameter *S*), and (ii) the theoretical treatment of singular topological defects and regions exhibiting pronounced biaxial character becomes typically ill-defined, as the director field alone lacks the necessary tensorial structure. Therefore, alternative approaches are developed based on **Q**-tensor^2,9,29,31^, which allows the scalar order parameter to vary continuously, thereby enabling a unified treatment of ordered and disordered states. This is particularly important in ferroelectric nematics, where the coupling between **P** and **Q** leads to the melting of the nematic order, directly influencing the stability of the polar phase. In our framework, the nematic and polar orders are treated as independent fields, with bulk elastic terms decoupled from each other. The polar field is coupled to the elasticity through the flexoelectric couplings. Similar treatment of the nematic and polar order parameters as independent variables has been employed in recent studies^53,55,56^, where the authors construct free energy for different mesophases. The compressive term, relevant to chiral polar helices^53^, is in our case not included as the present work concerns achiral bulk phases. One limitation of the work is that we have not included the effects of the depolarization fields^15,18,48^ explicitly. In the uniform bulk phase, the effects of the depolarization field are strongly suppressed by screening, and their primary influence manifests near interfaces or in confined geometries^9,23^. In the modulated phase, ∇ ⋅ **P** ≠ 0 generates bound charges and a corresponding depolarization field, which increases the effective splay constant^48^. Its inclusion would shift the equilibrium modulation period and the location of the phase boundaries, and is a natural direction for a more quantitatively accurate description.

### Phase diagram of bulk ferroelectric nematics

We calculate the phase diagram of bulk ferroelectric nematics by minimizing the total free energy, specifically adopting a two-dimensional description of the Q tensor formalism that still fully captures the relevant physics. The functional of free-energy is minimized in a periodic layer (typically 128 × 128 mesh points; for more, see “Methods”) to mimic the bulk ferroelectric nematic and incorporates local orientational order, defect formation, coupling effects, and elastic interactions, all of which are governed by in-plane fields. The two-dimensional framework provides a complete representation of the system while avoiding the methodological (and computational) complexity of a three-dimensional treatment, but notably limited by not capturing three-dimensional structures involving out-of-plane director distortions, twist deformations, defect-line escape into the third dimension, or spatial variations along the thickness of the sample, which may become relevant in confined geometries.

**Fig. 1:**
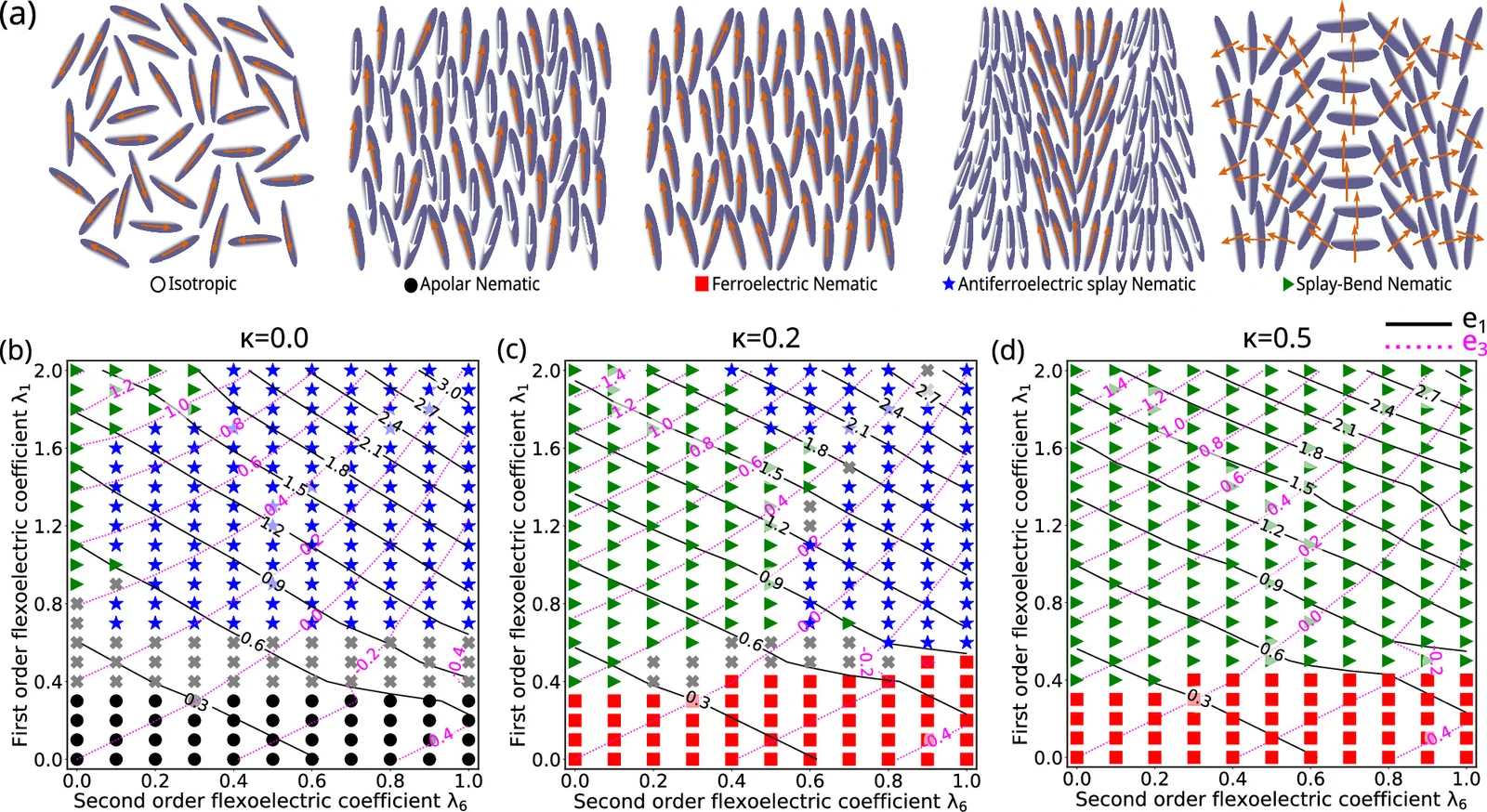
Phase diagram of bulk ferroelectric nematic liquid crystals. **a** A schematic of all main ferroelectric nematic phases. The orange (white) arrow represents the direction of **P** pointing in the up (down) direction. **b**–**d** Phase diagram for different flexoelectric anisotropies given by coupling coefficients *λ*_1_ and *λ*_6_, and for different polarization stiffness *κ*. Note the black and red contour lines, which give the flexoelectric anisotropy for splay *e*_1_ and bend *e*_3_ flexoelectric coefficients. Note how increasing polarization stiffness promotes the emergence of the splay bend phase, whereas increasing the splay flexoelectric coefficient *e*_1_ energetically favors the emergence of the splay phase. Crosses in (**b**, **c**) correspond to hysteretic metastable regions between the phases.

We identify five distinct phases of ferroelectric nematic liquid crystals, four of which are experimentally observed and one we predict in the regime of weakly orientationally coupled polarization and director. Figure 1a illustrates these phases: ferroelectric nematic, apolar nematic, antiferroelectric splay nematic, and splay-bend nematic phases. In addition to these, the isotropic phase (at high temperatures), in which both the nematic and polarization order parameters vanish, emerges as a trivial solution of the model. We numerically calculate phase diagrams for the effectively different flexoelectric anisotropies *λ*_1_ − *λ*_6_, and for different values of the polarization elasticity coefficient *κ* = 0.0, 0.2 and 0.5 (Fig. 1b–d), which we show are the major material parameters (properties) affecting the bulk ferroelectric nematic phases. Since these coefficients encode material-specific properties, different choices of *λ*_*i*_ and *κ* correspond to different ferroelectric nematic materials, and the phase diagrams thus map how the bulk behavior varies across the material family. The black (red) lines represent the values of the splay *e*_1_ (bend *e*_3_) flexoelectric coefficient in the director representation. The parameter *κ* governs the energetic penalty associated with spatial gradients in the polarization field **P**. For *κ* = 0, there is no energetic cost for local fluctuations in **P**, allowing the system to adopt a homogeneous but apolar nematic configuration when the flexoelectric couplings are weak. For nonzero values of *κ*, spatial variations in the polarization field **P** incur an energetic penalty, which favors the emergence of a uniform polarization and stabilizes the ferroelectric nematic phase. Furthermore, when there is a significant anisotropy between the effective flexoelectric coefficients *e*_1_ and *e*_3_, the system favors an antiferroelectric splay configuration. This phase is characterized by periodic modulations in the polarization field with sharp domain walls that exhibit Ising-like behavior. As *κ* increases, large spatial variations in **P** become energetically unfavorable, which leads to a redistribution of the distortions into bending modes of both the nematic and polarization fields. Consequently, the splay-bend nematic phase emerges. This change reflects the system’s tendency to accommodate polarization elasticity by adopting more smoothly varying, yet spatially modulated, polar textures. To the best of our knowledge, this phase has not yet been reported experimentally in FNLCs. Though there have been previous reports on analogues phases in other contexts, such as dimeric liquid crystals and colloidal systems^57–63^. We predict that lifting the co-alignment between the nematic director and the polarization field can give rise to a new splay-bend phase. Note that the angle between **P** and **n** is not imposed geometrically. It emerges self-consistently from the minimization of the free energy determined by the competition between flexoelectric and anisotropic coupling terms.

### Antiferroelectric nematic liquid crystals phase

**Fig. 2:**
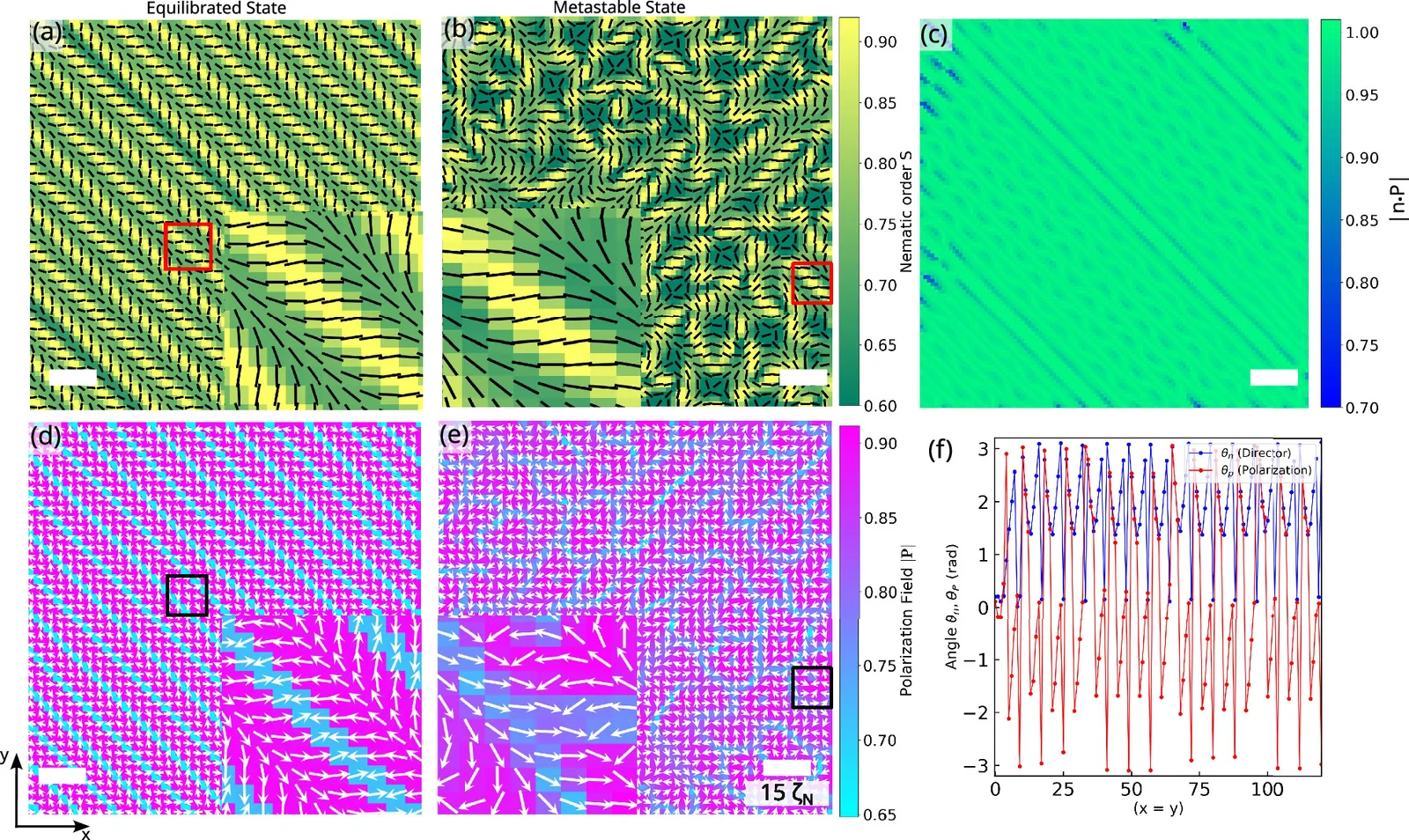
Antiferroelectric splay nematic phase. **a** Nematic and **d** polarization (right) morphologies indicating an antiferroelectric splay nematic liquid crystal phase (*η* = 0.02, *κ* = 0.2, *λ*_6_ = 0.8, *λ*_1_ = 1.8, *μ* = −1.0). The color bar represents *S* and |**P**| magnitudes, respectively. The black (white) arrows represent the orientation of the director (polarization) field. **b**, **e** Represents the metastable state for the same parameters. In **c**, the dot product ∣**n** ⋅ **P**∣ is plotted to identify the correlation between the director and the polarization vector. **f** Plot of angle *θ*_*n*_, *θ*_*P*_ with the *x*-axis along the diagonal (*x* = *y*), confirming Ising-like characteristics of domain walls in the polarization field. The scale bars correspond to 15 nematic correlation lengths. Source data are provided as a source data file.

Figure 2 illustrates the characteristic structure of the antiferroelectric splay nematic phase (for *η* = 0.02, *κ* = 0.2, *λ*_1_ = 1.8, and *λ*_6_ = 0.8). Panels (a) and (d) show the spatial profiles of the nematic scalar order parameter *S* and the polarization magnitude ∣**P**∣, respectively. The color bars represent the corresponding field magnitudes, while the black and white arrows denote the local orientation of the director **n** and the polarization vector **P**, respectively. The emergent structure reveals a spatially modulated polarization field with alternating domains, consistent with an antiferroelectric arrangement. The domain walls separating oppositely polarized regions are sharply defined and exhibit an Ising-like character. The corresponding director field shows weak modulation but remains overall aligned within each domain, indicating a strong coupling between the nematic and polar orders, which is consistent with the experimental observation of this phase^23^. The splay nematic phase exhibits a periodic, layer-like modulation of the director and polarization fields without an associated density modulation. The periodicity is not imposed a priori but emerges from the free-energy minimization through the balance between the flexoelectric couplings and the elastic terms. To quantify the correlation between the two fields, snapshot Fig. 2c presents the spatial profile of ∣**n** ⋅ **P**∣. The plot confirms a predominantly parallel or anti-parallel alignment of the director and polarization vectors within domains, while the domain boundaries mark a clear reversal in the polarization direction. Furthermore, we plot the director angle *θ*_*n*_ and the polarization angle *θ*_*P*_ along the diagonal direction. The results show that the polarization field undergoes a reversal at the domain boundaries. Also note that the polarization magnitude is inversely correlated with the nematic order in this phase, which arises from the coupling of **P** to gradients of **Q**, enhancing polarization in regions with the strong spatial variation of *S*. All these are direct characteristics of the antiferroelectric splay phase, stabilized in this regime of strong flexoelectric coupling and moderate polarization elasticity.

The demonstrated antiferroelectric phase proves to be very susceptible to the emergence of different topological defects in the periodic pattern, leading to various metastable structures. The well-defined periodic antiferroelectric splay nematic structures shown so far were generated using a perfectly periodic initial condition: the director field was set as $(\cos \theta,\sin \theta )$, with $\theta={\theta }_{0}\sin (x+y)$, and the polarization vector was aligned along the director. This seeded configuration introduces a spatially modulated orientation without imposing a fixed wavelength, allowing the system to spontaneously select its periodicity through competition between different coupling terms. This choice is motivated by a purely practical consideration. In contrast, simulations initialized from an isotropic or random local director state for both nematic and polarization fields frequently converged to metastable configurations populated by numerous defects, as shown in Fig. 2b, e. Sub-figures represent the nematic and polarization field for the same set of simulation parameters. These metastable states exhibit a partial splay character but lack long-range periodicity. We observe that the system can easily become trapped in these metastable states during its relaxation with free energy at the order of 10^−3^*L*/(*Δx*)^2^ higher relative to the fully periodic ground state. The bend distortion visible at the walls in this metastable state is not characteristic of the equilibrium antiferroelectric phase, where experimentally only Ising-type walls are observed^23^. Although these configurations retain some splay features, the high defect density obscures the underlying order. Interestingly, the sign and spatial distribution of the topological charges associated with the defects provide indirect evidence of the emergent phase structure. We also note that *S* depends indirectly on the flexoelectric coupling through its effect on equilibrium ∣**P**∣. Hence, the phase stabilized at higher *λ*_*i*_, also exhibits higher *S* compared to the uniform ferroelectric phase.

**Fig. 3:**
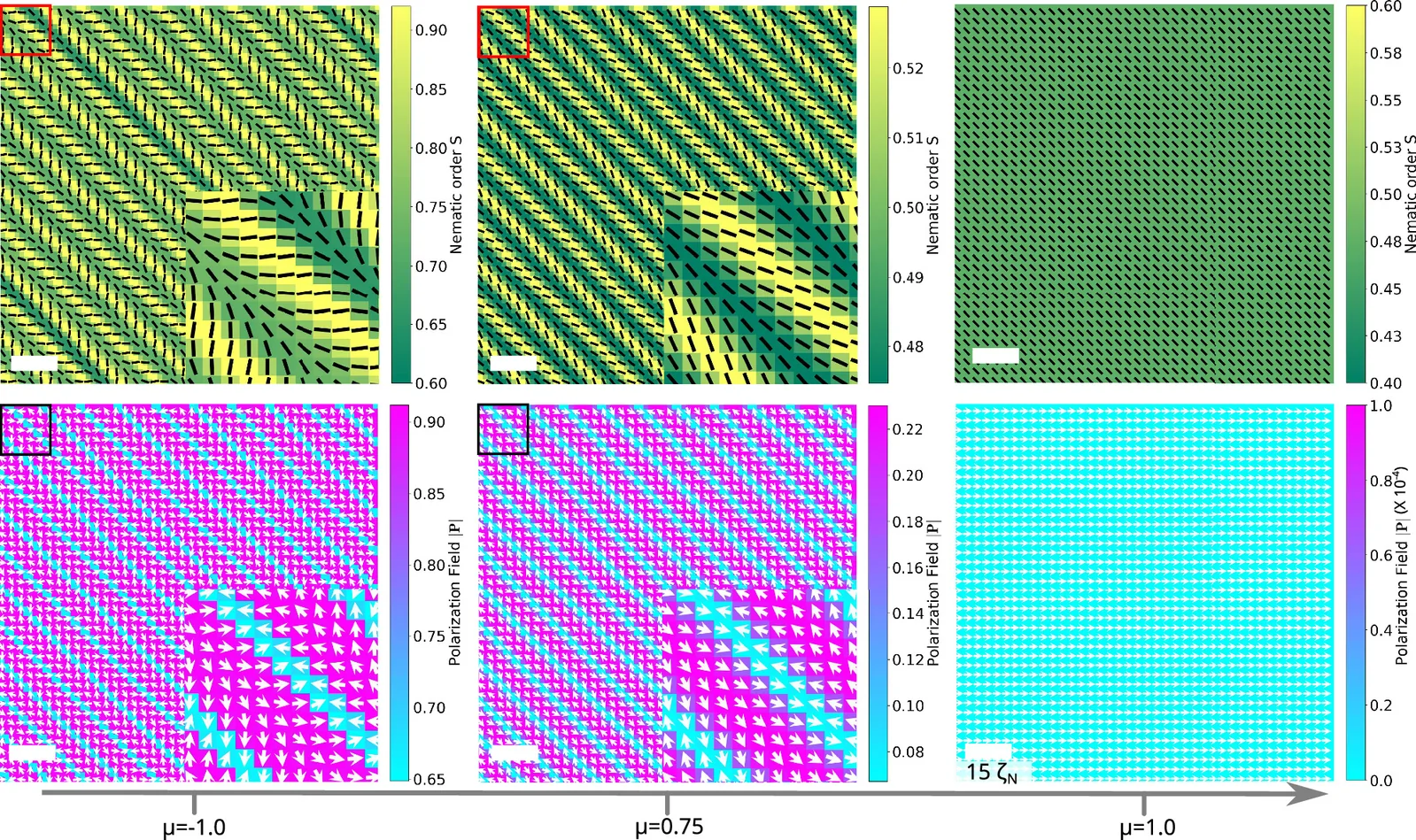
Effective variation of temperature in splay nematic phase. Nematic (top) and polarization (bottom) field for different values of *μ* (*η* = 0.02, *λ*_6_ = 0.8, *λ*_1_ = 1.8, *κ* = 0.2). These snapshots show that the splay phase first softens and then makes the transition to the apolar nematic phase as the temperature increases. The black (white) arrows represent the orientation of the director (polarization) field. The scale bars correspond to 15 nematic correlation lengths. Source data are provided as a source data file.

We demonstrate the influence of temperature on the stability of the splay nematic phase by varying the parameter *μ*, which effectively controls the temperature in our model. Figure 3 shows the resulting nematic (top) and polarization (bottom) morphologies for increasing values of *μ*. At lower *μ*, the splay modulation is well observed with distinct polarization domains. As *μ* increases, the modulations gradually weaken, indicating a softening of the splay structure. The modulated polarization vanishes entirely for large *μ* values, and the system relaxes into an apolar, uniform nematic phase. This transition underscores the thermal stability of the splay phase and directly implies the need for a sufficiently strong ferroelectric ordering (i.e., lower *μ*) for the emergence and stabilization of modulated polar structures. These observations confirm that the existence of the splay nematic phase critically depends on the system being in the ferroelectric regime^1,9^.

We also show that increasing the anisotropy in the flexoelectric coupling leads to a tighter modulation of the director and polarization fields, effectively reducing the characteristic length scale of the splay structure. Supplementary Fig. 2 presents the morphologies of the nematic field (top row) and the polarization field (bottom row) for increasing values of the first flexoelectric coefficient *λ*_1_ = 0.8, 1.2, 1.8 and for fixed second flexoelectric coefficient *λ*_6_ = 0.8. The snapshots clearly reveal that the spatial periodicity of the modulation decreases as *λ*_1_ increases. Since *λ*_6_ is constant, the observed reduction in periodicity can be attributed directly to the enhanced splay flexoelectric coefficient *e*_1_, as defined in Eqs. (4 and 5).

### Splay-Bend nematic liquid crystals phase

**Fig. 4:**
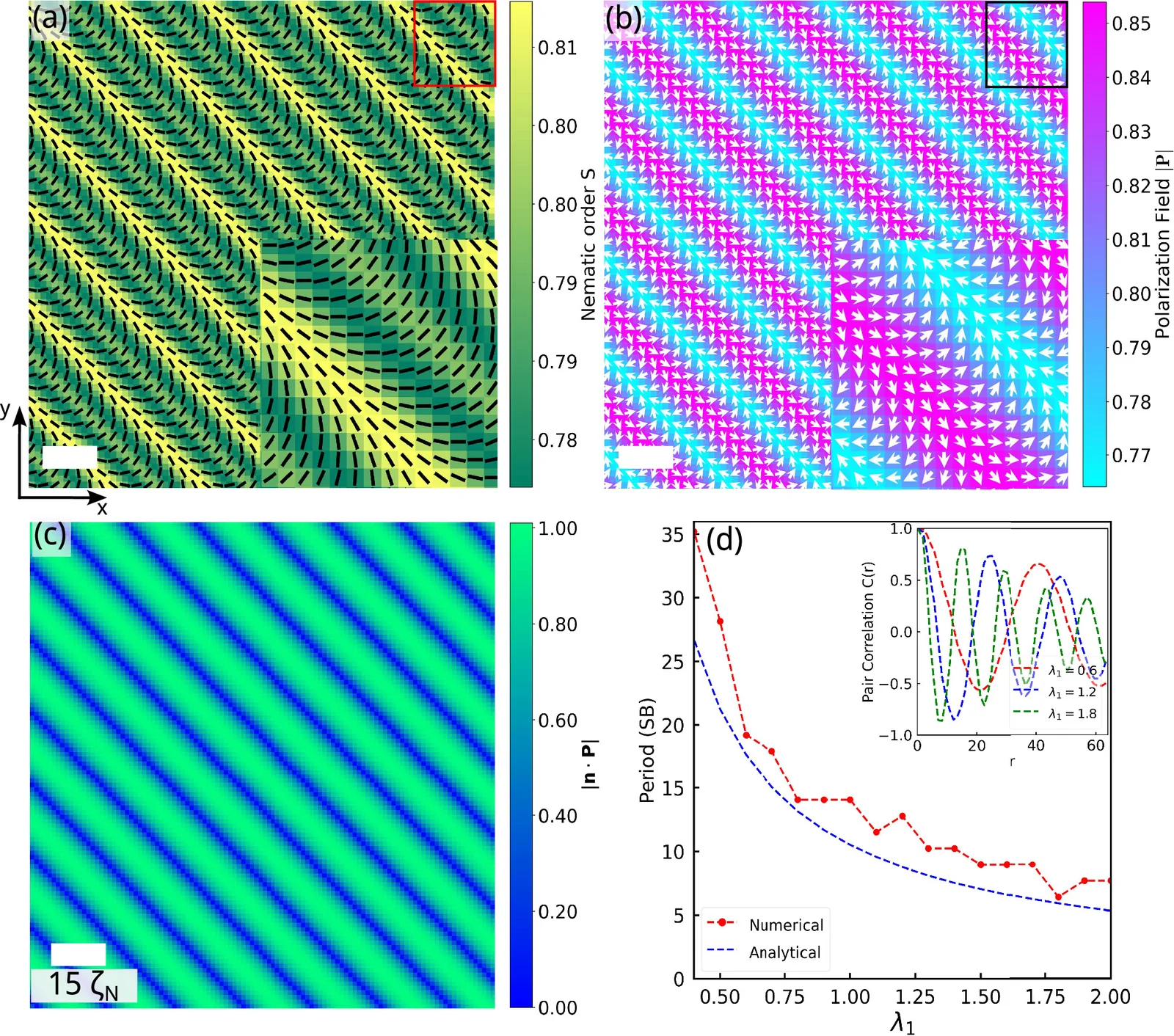
Splay-bend ferroelectric nematic phase. **a** Nematic. **b** Polarization (right) morphologies indicating a splay-bend nematic liquid crystal phase (*η* = 0.02, *κ* = 0.5, *λ*_6_ = 0.8, *λ*_1_ = 1.8, *μ* = −1.0). The color bar represents *S* and ∣**P**∣ magnitudes, respectively. The black (white) arrows represent the orientation of the director (polarization) field. **c** Gives the dot product ∣**n** ⋅ **P**∣ as an effective measure of the relative angle between the director and the polarization vector. **d** The plot depicts the periodicity of the splay-bend phase as a function of *λ*_1_, along with the analytically obtained values. In the inset, the pair correlation function of the nematic field is plotted for *λ*_1_ = 0.6, 1.2, 1.8. The scale bars correspond to 15 nematic correlation lengths. Source data are provided as a source data file.

Our approach enables the prediction of a novel ferroelectric nematic mesophase that we call the splay-bend phase and appears in our simulation for the regime of weakly orientationally coupled polarization and director. Figure 4 presents the steady-state morphology of the splay-bend ferroelectric nematic phase. Figure 4a, b depict spatial profiles of the nematic scalar order parameter *S* and the polarization magnitude ∣**P**∣, respectively. The black (white) arrows depict the local orientation of the nematic (polarization) field. We observe that the polarization field exhibits well-defined periodic modulations in both magnitude and direction, whereas the nematic director undergoes smooth bend and splay distortions in phase with the polarization field. To further characterize the coupling between the nematic and polar fields, in (c), we show the spatial variation of the dot product ∣**n** ⋅ **P**∣, which quantifies the local alignment between the two fields. Regions of reduced correlation correspond to locations where the polarization reverses or bends sharply with respect to the director, typically near inflection points of the modulation. Note that the relative orientation of the two fields alternates between parallel in the splay regions and perpendicular in the bend regions. Further, Fig. 4d shows the dependence of the periodicity of the modulations on the flexoelectric coupling coefficient *λ*_1_. The results clearly demonstrate that increasing *λ*_1_ leads to a shorter periodicity of the structure, reflecting a more tightly modulated splay-bend texture driven by enhanced flexoelectric interaction.

We also calculated (from free energy in Eq. (1)) the expected analytical expression (blue dotted line) for the dependence of the periodicity of this phase on the model parameters. Our analytical estimate is in good agreement with the numerical results. Moreover, the expression demonstrates that the characteristic periodicity of the splay-bend structure is set by an inverse dependence on the flexoelectric coefficient *λ*_1_. The inset in (d) shows the corresponding pair-correlation function of the director field, which is used to quantitatively extract the periodicity of the modulated phase. We should emphasize that, to the best of our knowledge, the splay-bend ferroelectric nematic phase predicted in our simulations has not yet been experimentally observed in conventional ferroelectric nematic materials. Our free energy modeling suggests that this is likely due to the relatively rigid coupling between the polarization vector **P** and the director field **n** in currently available compounds, i.e., the polarization **P** is in all materials roughly parallel along director field **n**, which constrains the system’s ability to accommodate the necessary spatial variation in relative orientation. Differently, our theoretical framework, within the full span of material parameters, allows for effectively independent variations in these fields, which in turn enables the stabilization of the splay-bend texture. As such, our results predict that in materials where the coupling between **n** and **P** could be more compliant, either intrinsically or through external tuning, the splay-bend phase is likely to emerge as a stable and dominant configuration.

The evolution of the splay-bend phase under increasing temperature, modeled through variations in the parameter *μ*, is presented in Supplementary Fig. 3. The snapshots present the steady-state configurations of the nematic (top row) and polarization (bottom row) fields for increasing temperature (i.e., increasing *μ*) and fixed other material parameters (*η* = 0.02, *λ*_6_ = 0.8, *λ*_1_ = 1.8, and *κ* = 0.5). Initially, the system exhibits a well-defined splay-bend morphology characterized by modulations in the director and polarization fields. As *μ* increases, the structure transitions into a splay-dominated configuration with antiferroelectric domains in the polarization field, marked by alternating regions of opposite polarization separated by domain walls. With further increase in temperature, the polar order vanishes entirely, and the system relaxes to an apolar, uniformly aligned nematic phase. This sequence of transitions again directly underscores the thermal sensitivity of modulated polar structures.

### Ferroelectric nematic liquid crystal phase

**Fig. 5:**
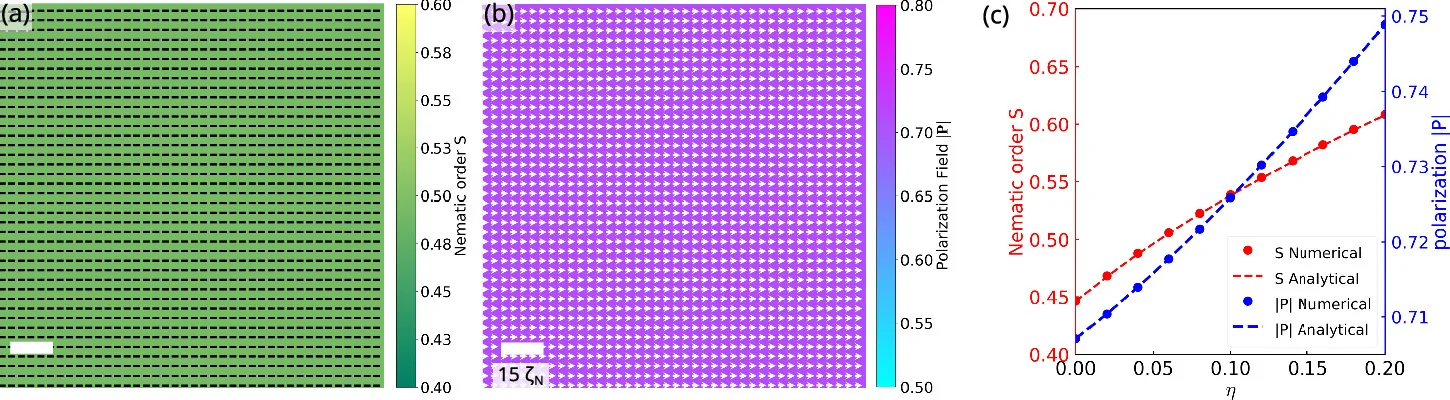
Ferroelectric nematic phase. **a** Nematic director. **b** Polarization (right) profiles for *η* = 0.02, *κ* = 0.2, *λ*_6_ = 1.8, *λ*_1_ = 0.2, indicating a uniform and co-aligned phase which is polar. The color bar represents *S* and ∣**P**∣ magnitudes, respectively. The black (white) arrows represent the orientation of the director (polarization) field. In **c**, magnitudes for *S* and ∣**P**∣ vs. *η* are plotted and show good agreement with the analytically calculated values tabulated in Table 1. The scale bars correspond to 15 nematic correlation lengths. Source data are provided as a source data file.

The uniform polar ferroelectric nematic phase is demonstrated in Fig. 5, at a selected representative point in the phase diagram (*η* = 0.02, *κ* = 0.2, *λ*_6_ = 1.8, *λ*_1_ = 0.2, *μ* = −1.0). Figure 5a shows the spatial distribution of the nematic scalar order parameter *S*, while (b) depicts the corresponding polarization magnitude ∣**P**∣. The system equilibrates into a homogeneous state where both *S* and ∣**P**∣ are spatially uniform, and the director and polarization fields are strictly co-aligned across the domain, forming a stable, fully ordered ferroelectric nematic phase with no elastic distortions. Notably, this phase is distinct from the apolar nematic, where the solution holds the up-down symmetries resulting in net ∣**P**∣ = 0 (as shown in Fig. 1b for *κ* = 0). In contrast, *κ* ≠ 0 stabilizes the ferroelectric nematic phase with a finite ∣**P**∣.

In the limit of homogeneous director and polarization, the homogeneous solutions for *S* and ∣**P**∣ can be calculated analytically and are provided in Table 1. The details of the solution can be found in Supplementary Note 3. In (c), we present the variation of the magnitudes *S* and ∣**P**∣ as functions of the dielectric anisotropic coupling parameter *η*. The numerical results are in excellent agreement with the analytically computed equilibrium values. These magnitudes increase monotonically as a function of *η*.

**Table 1:** Homogeneous fixed-point solutions for the nematic tensor and polarization fields

Order Parameter	Homogeneous Solutions
${Q}_{11}^{*}$	$$\begin{array}{c}-\frac{{2}^{1/3}\left(-2{\eta }^{2}-A\nu \right)}{{\left(27{C}^{2}\eta \mu {\nu }^{2}+\sqrt{729{C}^{4}{\eta }^{2}{\mu }^{2}{\nu }^{4}+108{C}^{3}{\nu }^{3}{\left(-2{\eta }^{2}-A\nu \right)}^{3}}\right)}^{1/3}}\\+\frac{{\left(27{C}^{2}\eta \mu {\nu }^{2}+\sqrt{729{C}^{4}{\eta }^{2}{\mu }^{2}{\nu }^{4}+108{C}^{3}{\nu }^{3}{\left(-2{\eta }^{2}-A\nu \right)}^{3}}\right)}^{1/3}}{3\cdot {2}^{1/3}C\nu }\end{array}$$
${Q}_{12}^{*}$	0
${P}_{1}^{*}$	$\sqrt{\frac{\mu+2\eta {Q}_{11}^{*}}{\nu }}$
${P}_{2}^{*}$	0

The table lists the homogeneous fixed-point solutions of the nematic tensor and polarization fields. The left column specifies the field variables, and the right column gives the corresponding homogeneous solutions.

## Discussion

In this work, we demonstrate bulk analysis of ferroelectric nematic liquid crystal phases, providing mesoscopic insight into the structure of observed nematic ferroelectric phases-ferroelectric nematic, antiferroelectric splay nematic, apolar nematic, and isotropic phase- and also predict a possible new splay-bend phase, if the orientational bond between the nematic director and polarization could be energetically reduced. We construct a Landau-de Gennes-type free energy framework that appropriately models the mesophase structures as well as the phase sequence, based on incorporating nematic (**Q**) and polarization (**P**) order parameters and their mutual couplings. Specifically, our study revealed the existence of five distinct phases, including uniform polar nematics, antiferroelectric splay nematics, and modulated splay-bend structures, which arise from the competition between elastic, polar, and flexoelectric interactions. The morphologies are strongly influenced by the polarization elasticity, where incorporating anisotropy in the flexoelectric coupling proves crucial to reproduce the experimentally observed phase behavior. We further demonstrate how temperature-dependent materials parameters, such as the polarization magnitude parameter *μ*, drive transitions from modulated to homogeneous or apolar phases, as a direct route to tune the behavior of FNLCs. We also highlighted that ferroelectric nematics -especially in splay and splay-bend phases- are inherently susceptible to the formation of various defect-populated metastable structures, notable to the point of breaking the long-range periodicity of the structure (such as splay pitch). The phenomenology of FNLCs is very rich with phases reported in the literature-such as the chiral ferroelectric nematic, the twist-bend ferroelectric (*N*_*TBF*_), the *N*_*X*_ mesophase, and ferroelectric smectic phases^12,19,24–28,52^. In addition, the framework can be naturally extended to confined FNLC systems by including surface anchoring and surface-originating electrostatic contributions. These represent natural directions of future extension of the work.

A notable strength of our complete free energy approach is that it provides direct access to the scalar order parameter *S*, which is directly linked to experimentally measurable quantities such as birefringence and density. Distinctly, the variation of *S* arises self-consistently from the minimization of the free energy as a function of the coefficient *μ*, which in an experimental context can be associated with temperature. This naturally establishes a theoretical connection to birefringence measurements, where the slope of birefringence versus temperature is known to change at the nematic-splay-nematic transition. Density provides an additional route for comparison: in ferroelectric nematics, orientational order and density are strongly correlated, and one may assume *Δρ* ∝ *S*^2^ to leading order. This connects our theory directly to X-ray scattering, since the scattering intensity is proportional to the square of the Fourier transform of the density modulation, which in turn is governed by the spatial variation of *S*. In particular, experiments have shown that the density in the antiferroelectric splay nematic phase exhibits a pronounced pretransitional behavior near the nematic transition^64^. Within our model, this raises the natural question whether analogous anomalies can be inferred from the behavior of the average order parameter 〈*S*〉. As illustrated in Supplementary Fig. 4, the variation of 〈*S*〉 with *μ* reveals a sharp transition *N*_*S*_ → *N* phase change. Along this trajectory, *S* increases from the nematic to the splay-nematic to the ferroelectric phase, reflecting the mutual coupling of polar and orientational order in the free energy. We present this as a qualitative feature of the model rather than a quantitative comparison to a specific material. To generalize, the *Q*-tensor description presented here thus provides not only a systematic framework for coupling between polarization and orientational order, but also a powerful tool for interpreting birefringence and scattering data across the sequence of phase transitions.

Notably, the model also predicts the emergence of a splay-bend phase in which the director **n** and polarization **P** fields are not rigidly aligned and can evolve independently. In such cases, the presence of a finite angle between the two fields naturally gives rise to biaxial ordering. Given that biaxial nematic phases have been elusive in experiments, this theoretical pathway offers a promising direction for the design of liquid crystalline materials that are both ferroelectric and biaxial. The coexistence of polar and biaxial order in such systems could enable novel textures and tunable electro-optic properties, broadening the functional scope of soft matter systems. In this emerging area of research on FNLCs, combined experimental and theoretical efforts are needed to understand the fundamentally rich and technologically important properties. Our work is a timely and modest step in this direction.

## Methods

We introduced a mesoscopic model to capture the bulk mesophases of FNLCs. Such an approach has advantages in its ability to capture complex ordering phenomena using symmetry principles and a minimal set of physically motivated assumptions without worrying about detailed molecular-scale information. It can incorporate not only the thermodynamic aspects of symmetry breaking, but also spatial inhomogeneities and defect structures through gradient terms. Moreover, the formalism is easily extensible to include multiple interacting fields, external fields, and dynamic effects, making it versatile for applications. Such frameworks are also computationally efficient, offering a practical alternative to atomistic simulations, which require detailed input on molecular geometry and interaction forces. The order parameters for FNLCs are the **Q**-tensor and polarization field **P**. The **Q**-tensor is defined in terms of the director **n** and scalar order parameter *S* as *Q*_*ij*_ = *S*(*n*_*i*_*n*_*j*_−*δ*_*ij*_/*d*). Here, $S=\overline{{P}_{2}(\cos \theta )}$, where *θ* is the angle between the long axis of a molecule and the director **n**, and the overbar denotes an average over all molecules. Here, *P*_2_(*x*) is the second Legendre polynomial. In the isotropic phase, where molecular orientations are random, *S* = 0; on the contrary, *S* = 1 corresponds to a perfect alignment along the director, indicative of an ideal uniaxial nematic phase. The second order parameter **P** breaks the inversion symmetry, providing a direction to the polarity of the system. In this work, we restrict ourselves to strictly two-dimensional (*d* = 2) systems.

In the numerical minimization, we use a 2D **Q**-tensor formalism as it still captures the leading phenomenology of ferroelectric nematics. In *d* = 2, **Q**-tensor has two independent components,

6 $${{\bf{Q}}}=\left(\begin{array}{cc}{Q}_{11}&{Q}_{12}\\ {Q}_{12}&-{Q}_{11}\\ \end{array}\right),$$

Similarly, the polarization field can be written as ${{\bf{P}}}={P}_{1}\hat{x}+{P}_{2}\hat{y}$. The values of director **n** and *S* can be obtained by equating Eq. (6) with the definition of the **Q**-tensor. To determine the equilibrium configuration of the system, we employ a purely relaxation dynamics governed by the time-dependent Ginzburg-Landau (TDGL) formalism. The evolution of the fields is described by a set of coupled differential equations of the form

7 $$\frac{\partial \psi }{\partial t}=-{{{\Gamma }}}_{\psi }\,\frac{\delta f[{{\bf{Q}}},\,{{\bf{P}}}]}{\delta \psi }.$$

where *ψ* represents either the nematic order parameter **Q** or the polarization field **P**, and *δG*/*δψ* denotes the functional derivative of the total free energy *f*[**Q**, **P**] with respect to the corresponding field. The mobility coefficients Γ_*ψ*_ are set to unity for simplicity, unless otherwise specified. This relaxation dynamics ensures that the system evolves toward a local or global minimum of the free energy, thereby capturing the relaxation processes associated with domain formation and coarsening phenomena. We solve these equations in units of mesh resolution *Δx*, tensorial elastic constant *L*, and rotational viscosity Γ. We numerically solve the equations using an isotropic Euler discretization of the *N* = 128^2^ system size. The boundary conditions are chosen to be periodic to simulate the bulk behavior and remove edge effects. We take a temporal mesh size *Δt* = 0.01(*Δx*)^2^/Γ*L* such that the simulations are stable. The numerical stability of the solutions with respect to the grid size and time step has been depicted in Supplementary Fig. 1. Tr**Q**^3^ is zero in two dimensions. The values of other model parameters are: *A* = −0.483*L*/(*Δx*)^2^, *C* = 8.625*L*/(*Δx*)^2^, *μ* = −1.0*L*/(*Δx*)^2^, *ν* = 2*L*/(*Δx*)^2^. These are the standard values typically taken for the simulations of a typical nematic phase. The SI values of these parameters, along with typical values of the others, have been detailed in Supplementary Note 2. All statistical results presented here are averaged over 10 independent initial conditions, unless stated otherwise. Refer to Supplementary Note 1 and 2 for more details.

## Supplementary information

Supplementary Information

Transparent Peer Review file

## Data Availability

Source data has been deposited in the Figshare database under accession code: https://doi.org/10.6084/m9.figshare.32334291

## Code availability

The calculations can be done following the instructions in Methods and Supplementary Note 1 and 2. The code related to this study is available upon request.
