# Supplementary material for: Bulk mesophases modeling of ferroelectric nematic liquid crystals
Source: Nat Commun. 2026 Oct 3;17:11318. doi: 10.1038/s41467-026-78039-1 (PMC13724716; doi:10.1038/s41467-026-78039-1)
Supplement: Supplementary file 1 [file 41467_2026_78039_MOESM1_ESM.pdf]

Bulk Mesophases modeling of Ferroelectric Nematic Liquid Crystals

Aditya Vats

Faculty of Mathematics and Physics, University of Ljubljana, Ljubljana, Slovenia

Alenka Mertelj

Jožef Stefan Institute, Ljubljana, Slovenia

Miha Ravnik*

Faculty of Mathematics and Physics, University of Ljubljana, Ljubljana, Slovenia and Jožef Stefan Institute, Ljubljana, Slovenia

SUPPLEMENTARY NOTE 1: NUMERICAL METHOD

The free energy in Eq. (1) is used in Eq. (7) to obtain the energy minimization equations. These equations are integrated numerically with the standard Euler discretization method, where the first and second-order derivatives of a function $f(x,t)$ are discretized as:

$$\frac{\partial f}{\partial t} = \frac{f(t+\Delta t) - f(t)}{\Delta t}, \tag{1}$$

$$\frac{\partial f}{\partial x} = \frac{f(x+\Delta x) - f(x-\Delta x)}{2\Delta x}, \tag{2}$$

$$\frac{\partial^2 f}{\partial^2 x} = \frac{f(x+\Delta x) + f(x-\Delta x) - 2f(x)}{(\Delta x)^2}. \tag{3}$$

Here, $\Delta x = 1$ and $\Delta t = 0.01$ are chosen such that the solutions are stable and adequately capture the defect regions. As long as these conditions are met, the choice of these parameters does not change the nature of the solutions. We have also done the convergence test for a typical antiferroelectric splay nematic phase as a representative example, as reported in the Supplementary Table 1. The periodicity spans a different number of grid cells at each resolution, but the corresponding periodicity $\Lambda = \text{(period in cells)} \times \Delta x$ is unchanged. These values are obtained after the free energy has converged; a representative relaxation is shown in Supplementary Figure 1. This confirms that the reported phase structures and periodicity are numerically converged. The boundary conditions are chosen to be periodic in order to reduce the finite size effects and simulate the bulk behavior. Other parameter values and initial conditions used in the simulation are mentioned in the discussion following associated figures. These specifications are sufficient to reproduce all the results mentioned in the manuscript using any standard finite-difference implementation.

Δx	Δt	Grid	Period (cells)	Period ($\times\Delta x$)
Varying grid spacing ($\Delta t = 0.01$)				
1.0	0.01	128^2	11	11
0.5	0.01	256^2	22	11
0.25	0.01	512^2	44	11
Varying time step ($\Delta x = 1$)				
1.0	0.01	128^2	11	11
1.0	0.005	128^2	11	11
1.0	0.001	128^2	11	11

Supplementary Table 1. Numerical convergence for the antiferroelectric splay phase as a representative example. The upper block varies the grid spacing Δx at fixed time step and physical system size; the lower block varies the time step Δt at fixed grid. All these results in the same structure and periodicity pointing towards the numerical stability for grid and time step chosen.

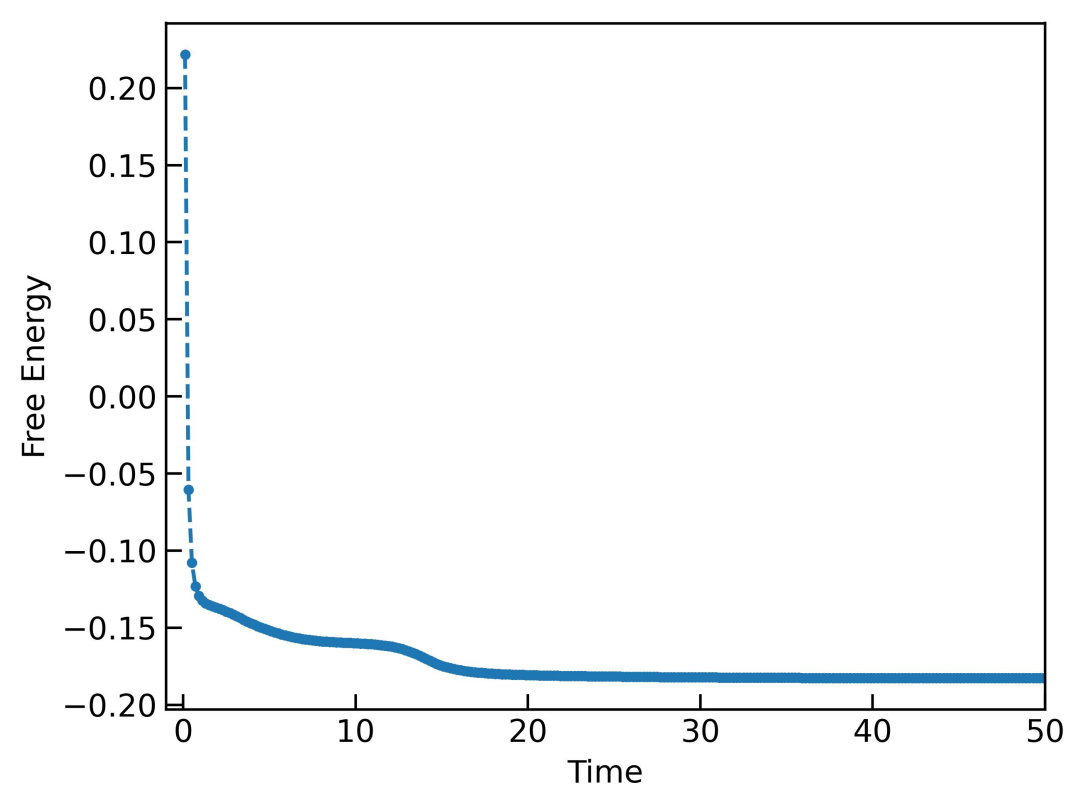

Supplementary Figure 1. Plot depicting numerical convergence of the free energy to a flat trajectory. The parameters are $\eta = 0.02, \lambda_6 = 0.8, \lambda_1 = 1.7, \kappa = 0.2$.

SUPPLEMENTARY NOTE 2: DIMENSIONLESS UNITS

The free energy minimization is obtained by expressing all quantities in dimensionless form using three reference scales drawn from the physics of the problem, nematic elastic constant L, the nematic correlation length ξ_N and the rotational viscosity γ. These fix the units of the energy, length and time respectively. The lengths are measured in units of ξ_N, energy densities in units of L/ξ_N^2. Setting $L = \xi_N = \gamma = 1$ makes every quantity dimensionless. The nematic order parameter $\mathbf{Q}$ is dimensionless by construction, and the polarization $\mathbf{P}$ is expressed in units of a reference value P_0 corresponding to the saturated polarization, so that it too is dimensionless.

The Landau coefficients (A, C, μ, ν) and the anisotropic coupling η are then given in units of the energy density L/ξ_N^2, while the elastic coefficients (L, κ) and the flexoelectric couplings (λ_i), which multiply gradient terms, carry the corresponding factors of ξ_N (see Supplementary Table 2). This is a standard choice for nematic systems. Note that the equilibrium structures reported here depend only on the length and energy-density scales. The viscosity enters only through the relaxational dynamics and controls how quickly the system settles into equilibrium, but not the state itself. We keep it here only so that the simulation time step can be given a physical value. The conversion to SI units is provided in Supplementary Table 2, corresponding to the measured representative values of RM734.

Quantity	Symbol	Simulation	SI value
Reference scales			
Length	ξ_N	1	3.7 nm
Grid spacing	$\Delta x = 1.5\,\xi_N$	1.5	5.6 nm
Elastic scale	L	1	3 pN
Energy density	L/ξ_N^2	1	2.2×10^5 J/m^3
Rotational viscosity	γ	1	0.1 Pa s
Time	$\gamma\xi_N^2/L$	1	0.46 μs
Time step	Δt	0.01	4.6 ns
Apolar coefficients			
	A	−0.483	-1.1×10^5 J/m^3
	C	8.625	1.9×10^6 J/m^3
Nematic elastic	L	1	3 pN
Polar coefficients			
	μ	−1.0	-2.2×10^5 J/m^3
	ν	2.0	4.4×10^5 J/m^3
Polar stiffness	κ	0.2	0.6 pN
Coupling coefficients			
Anisotropic coupling	η	0.02	4.4×10^3 J/m^3
Flexoelectric	λ_1	1.8	1.5 mN/m
Flexoelectric	λ_5	0	0
Flexoelectric	λ_6	0.8	0.65 mN/m

Supplementary Table 2. Correspondence between simulation and SI units, using following measured parameters of RM734: splay elastic constant $L \approx K_1 \approx 3$ pN [1, 2], correlation length $\xi_N \approx 3.7$ nm (approximate), rotational viscosity $\gamma \approx 0.1$ Pa s [3]. These reference values are taken from measurements of RM734 and a different reference material is accommodated by rescaling L, ξ_N, and γ.

In Supplementary Table 3, we summarize the free-energy coefficients of Eq. (1), the physical role each plays, and possible experimentally accessible quantity which are used to measure these in standard nematic systems. Although the coefficients are not measured directly, each is linked to a standard experimental observable, which fixes its physically reasonable range. The apolar coefficients A and C are set by the isotropic–nematic transition temperature and the temperature dependence of the scalar order parameter, obtained from birefringence and calorimetry; the nematic elastic coefficient L corresponds to the Frank constants measured in Frederiks-threshold experiments. The polar coefficients μ and ν are fixed by the spontaneous polarization $|\mathbf{P}|(T)$ measured by polarization-reversal and pyroelectric currents, and the polar stiffness κ by the width of polar domain walls imaged by second-harmonic-generation (SHG) microscopy. The anisotropic coupling η is constrained by the equilibrium orientation of $\mathbf{P}$ relative to the director, and the flexoelectric couplings λ_i by their renormalization of the splay and bend elastic constants and by the resulting modulation period.

Coefficient	Physical role	Experimental observable	Ref.
A, C	Apolar Landau coefficients	Birefringence $\Delta n(T)$, T_{NI}, latent heat (DSC)	[4, 5]
L	Nematic elastic constant	Frank constants (Frederiks threshold)	[1, 6]
μ, ν	Polar Landau coefficients	Spontaneous polarization $|\mathbf{P}|(T)$	[3, 7]
κ	Polar stiffness	Polar domain-wall width (SHG), correlation length	[8, 9]
η	Anisotropic coupling	Equilibrium angle between $\mathbf{P}$ and $\mathbf{n}$ (SHG interferometry)	[3]
$\lambda_1, \lambda_5, \lambda_6$	Flexoelectric couplings	Renormalized splay/bend constants; modulation period	[1, 10]

Supplementary Table 3. Free-energy coefficients of Eq. (1), their physical role, and possible experimentally accessible quantities that constrain them.

SUPPLEMENTARY NOTE 3: HOMOGENEOUS FIXED POINT SOLUTIONS

Homogeneous stationary solutions $(\mathbf{Q}^*, \mathbf{P}^*)$ of the model correspond to spatially uniform configurations in which the system has relaxed to equilibrium. They are obtained by setting all spatial derivatives in the dynamical equations to zero, so that the gradient (elastic and flexoelectric) terms drop out and only the bulk Landau contributions remain. The resulting stationary conditions are purely algebraic and determine the bulk values of the order parameters. This yields four algebraic

equations:

$$AQ^*_{11} - C({Q^*_{11}}^2 + {Q^*_{12}}^2)Q^*_{11} + \eta(P^{*2}_1 - P^{*2}_2) = 0, \tag{4}$$

$$AQ^*_{12} - C({Q^*_{11}}^2 + {Q^*_{12}}^2)Q^*_{12} + \eta(2P^*_1 P^*_2) = 0, \tag{5}$$

$$\mu P^*_1 - \nu|\mathbf{P}|^2 P^*_1 + 2\eta(Q^*_{11}P^*_1 + Q^*_{12}P^*_2) = 0, \tag{6}$$

$$\mu P^*_2 - \nu|\mathbf{P}|^2 P^*_2 + 2\eta(Q^*_{12}P^*_1 - Q^*_{11}P^*_2) = 0, \tag{7}$$

These equations are coupled through the anisotropic term η, which links the nematic and polar order parameters. $Q^*_{11} = Q^*_{12} = P^*_1 = P^*_2 = 0$ trivially satisfies these equations and corresponds to the disordered isotropic state where both nematic and polarization order parameters are zero. For a uniform polar solution, without the loss of the generality, one can assume the solution to be pointing in the x-direction. This simplifies the above equations to

$$AQ^*_{11} - C({Q^*_{11}}^2)Q^*_{11} + \eta P^{*2}_1 = 0, \tag{8}$$

$$\mu P^*_1 - \nu P^{*2}_1 P^*_1 + 2\eta Q^*_{11}P^*_1 = 0, \tag{9}$$

These equations now correspond to two sets of solutions. With $P^*_1 = 0$, the system decouples and generates a apolar nematic solution with no coupling impact and $P^*_1 \neq 0$ choice leads to the polar magnitude in terms of the nematic order. Elimination of the P^*_1 yields a single cubic equation for nematic order. The physically relevant root of this equation together with the expression of P^*_1 defines the uniform ferroelectric fixed point. The η-dependent terms encode the coupling between the polar and nematic order, and scales the equilibrium values of the order parameters. The solutions derived above are summarized in Table 1 of the main text.

SUPPLEMENTARY NOTE 4: SUPPLEMENTARY FIGURES

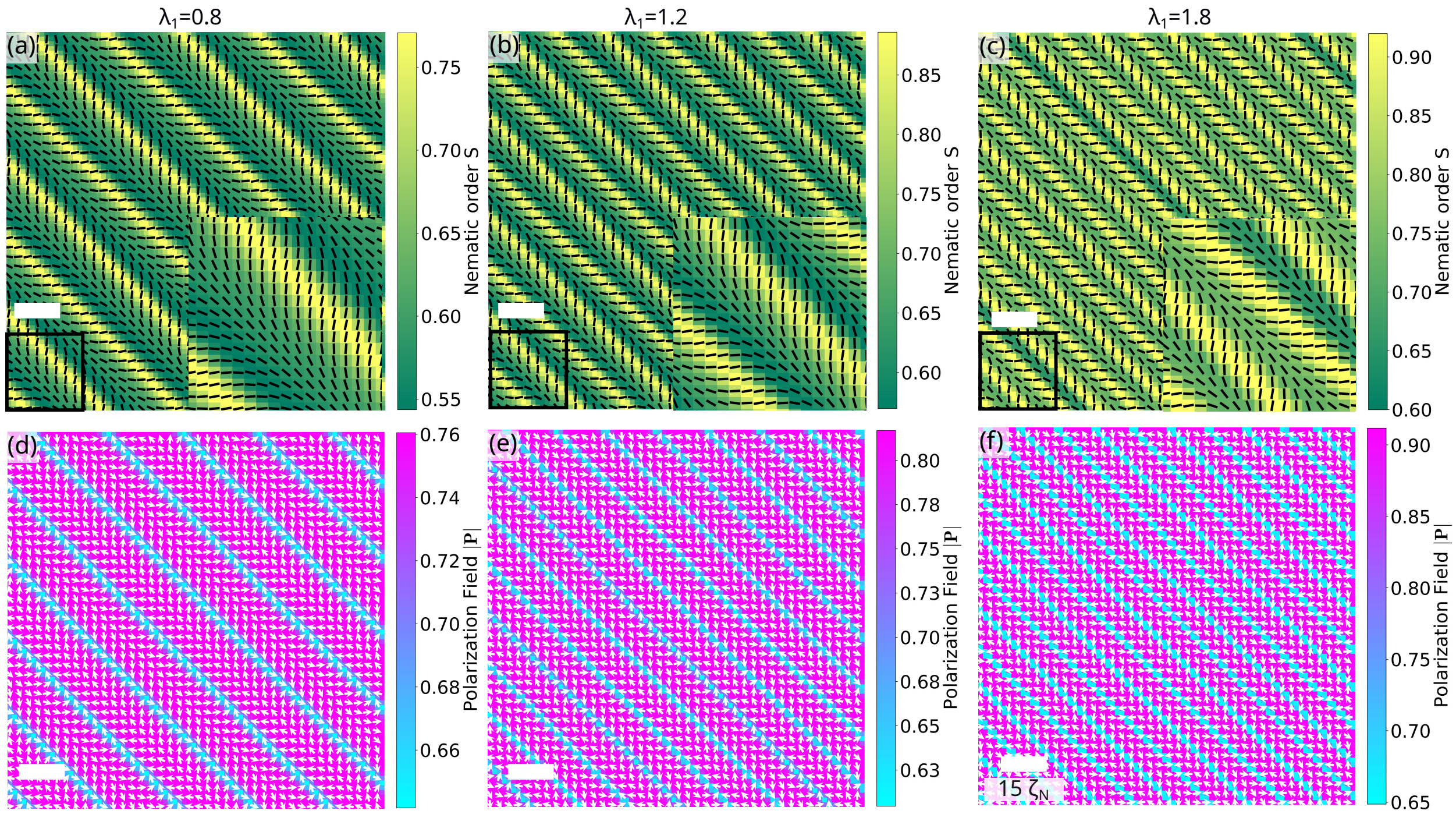

Supplementary Figure 2. Periodicity of splay nematic phase. Nematic (top row) and polarization (bottom row) morphologies illustrating the antiferroelectric splay nematic phase for $\eta = 0.02$, $\kappa = 0.2$, and $\lambda_6 = 0.8$, shown for increasing values of $\lambda_1 = 0.8,\ 1.2,\ 1.8$. The sequence highlights the reduction in modulation periodicity with increasing λ_1.

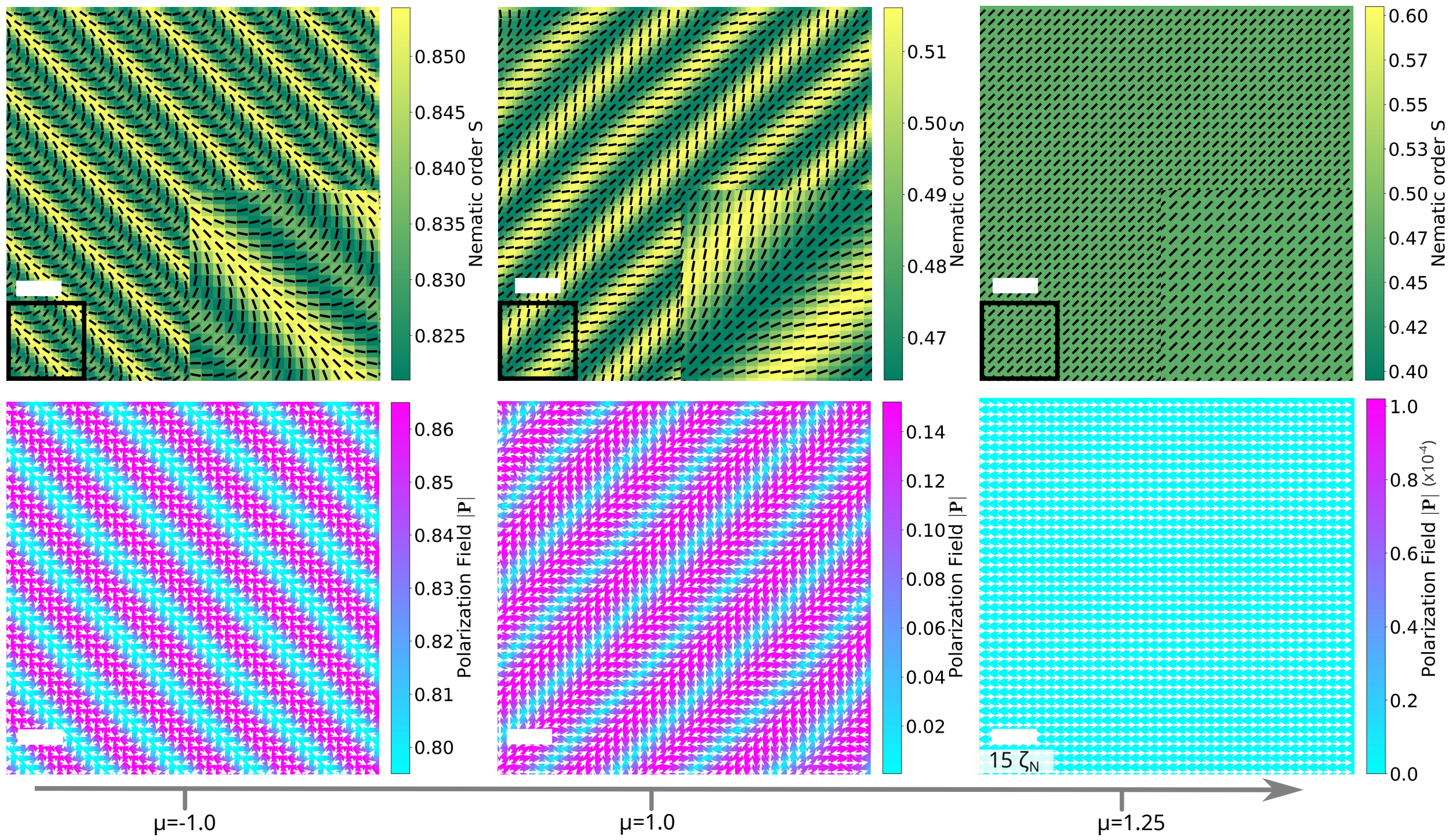

Supplementary Figure 3. Effect of μ on splay-bend phase. Nematic (top) and polar (bottom) field for different values of μ. Other parameters are $\eta = 0.02, \lambda_6 = 0.8, \lambda_1 = 1.8, \kappa = 0.5$. These snapshots show that the splay-bend phase first transitions to an antiferroelectric nematic liquid crystal and then makes the transition to the apolar nematic phase as the μ temperature increases.

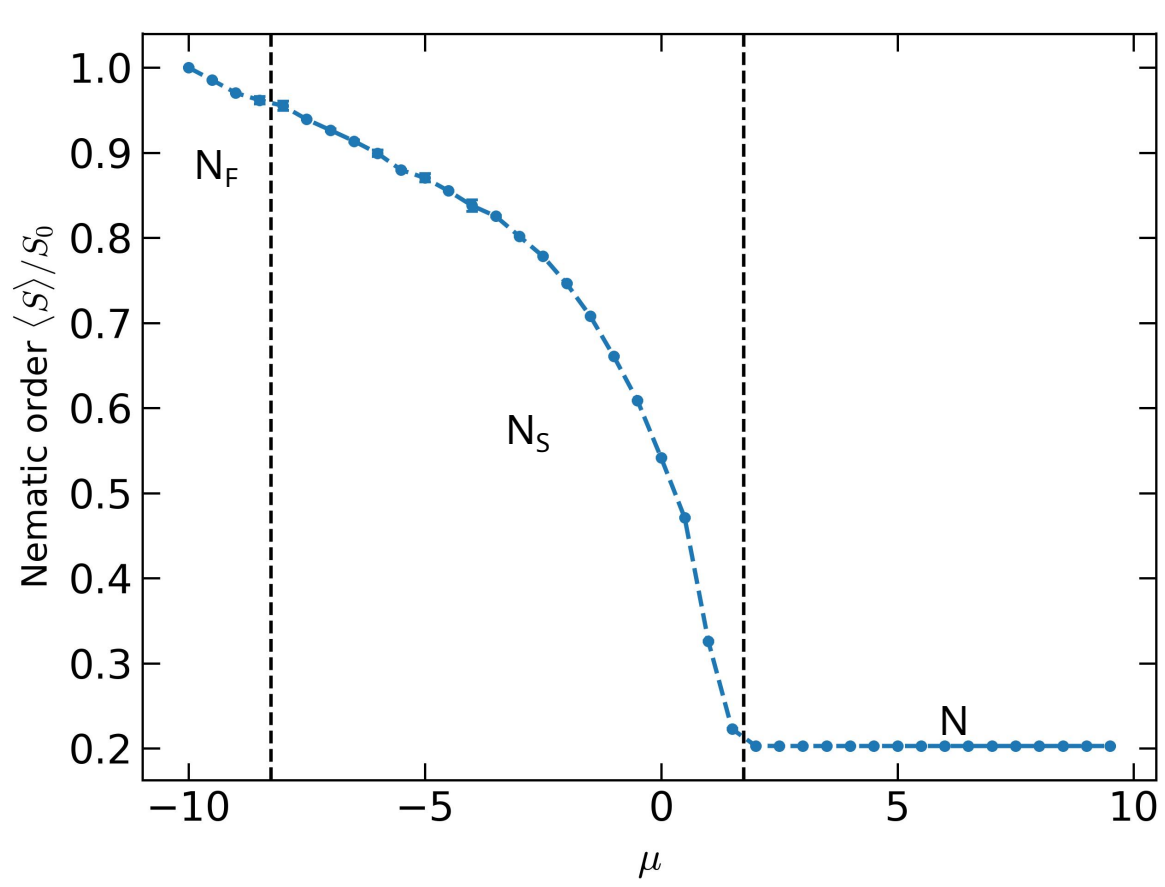

Supplementary Figure 4. Normalized average nematic order parameter $\langle \mathcal{S} \rangle / S_0$ vs. μ depicting the transition behavior from ferroelectric to antiferroelectric splay nematic to nematic phase transition. S_0 is the equilibrated value at $\mu = -10$. The points in the graph are the measured values and dashed line is a guide to the eyes. Other parameters are $\eta = 1.0, \lambda_6 = 0.8, \lambda_1 = 1.7, \kappa = 1.0$.

[1] N. Sebastián, M. Čopič, and A. Mertelj, Ferroelectric nematic liquid-crystalline phases, Phys. Rev. E **106**, 021001 (2022).

[2] A. Mertelj, L. Cmok, N. Sebastián, R. J. Mandle, R. R. Parker, A. C. Whitwood, J. W. Goodby, and M. Čopič, Splay nematic phase, Phys. Rev. X **8**, 041025 (2018).

[3] X. Chen *et al.*, First-principles experimental demonstration of ferroelectricity in a thermotropic nematic liquid crystal: Polar domains and striking electro-optics, Proc. Nat. Acad. Sci. **117**, 14021 (2020).

[4] P. G. de Gennes and J. Prost, *The Physics of Liquid Crystals* (Oxford University, Oxford, 1995).

[5] M. J. Stephen and J. P. Straley, Physics of liquid crystals, Rev. Mod. Phys. **46**, 617 (1974).

[6] F. C. Frank, I. liquid crystals. on the theory of liquid crystals, Discussions of the Faraday Society **25**, 19 (1958).

[7] J.-T. Pan *et al.*, Nonlinear geometric phase coded ferroelectric nematic fluids for nonlinear soft-matter photonics, Nat. Comm. **15**, 8732 (2024).

[8] N. Sebastián, L. Cmok, R. J. Mandle, M. R. de la Fuente, I. Drevenšek Olenik, M. Čopič, and A. Mertelj, Ferroelectric-ferroelastic phase transition in a nematic liquid crystal, Phys. Rev. Lett. **124**, 037801 (2020).

[9] P. Medle Rupnik, E. Hanžel, M. Lovšin, N. Osterman, C. J. Gibb, R. J. Mandle, N. Sebastián, and A. Mertelj, Antiferroelectric order in nematic liquids: flexoelectricity versus electrostatics, Adv. Sci. **12**, 2414818 (2025).

[10] C. Meyer, C. Blanc, G. R. Luckhurst, P. Davidson, and I. Dozov, Biaxiality-driven twist-bend to splay-bend nematic phase transition induced by an electric field, Science Advances **6**, eabb8212 (2020).
