# Supplementary material for: Bulk mesophases modeling of ferroelectric nematic liquid crystals
Source: Nat Commun. 2026 Oct 3;17:11318. doi: 10.1038/s41467-026-78039-1 (PMC13724716; doi:10.1038/s41467-026-78039-1)
Supplement: Supplementary file 2 [file 41467_2026_78039_MOESM2_ESM.pdf]

nature portfolio

Peer Review File

Bulk Mesophases Modeling of Ferroelectric Nematic Liquid Crystals

Corresponding Author: Professor Miha Ravnik

This file contains all reviewer reports in order by version, followed by all author rebuttals in order by version.

Version 0:

Reviewer comments:

Reviewer #1

(Remarks to the Author)
The paper by Vats, Mertelj and Ravnik is an exrteemly interesting development of a free energy model which expands our simplified model of flexoelectric contributions to something more complete. It is excellently written and presented with only a couple of exceptions. I suggest only minor changes would be needed to make this an excellent contribution to Nature Communications.

The introduction discusses developments to the physics and theory fairly completely. I would say an omission that could be rectified is the inclusion of structure property developments from a chemistry perspective. Particularly in light of their predictive design comments in the abstract. In particularly I believe Phys. Rev. E 104, 014704 (10.1103/PhysRevE.104.014704) should be included as this involved a simple model that has had by fair the biggest impact around predictive development in this space and possibly a few on the development of structure properties relationships in the synthetic space. Of course this is just my opinion and I leave up to the authors.

This is quite a minor point but the schematic of the Ns phase is not ideal in my opinion. My understanding is that the Ising walls should be vertical i.e. parallel to the director rather here they are cantered to each other leading to a narrowing and widening of the domains.

How do the authors include the alignment of the dipole and molecular long axis. Presumably the director n is coupled to the long axis orientation while the polarisation P is couple to the dipole orientation. The authors allude to some angle typically being small (reasonable) but then draw the splay bend phase using a dipole angle of 90 degrees. Could the authors comment more clearly on how they defined this angle in their calculations?

“For κ = 0, there is no energetic cost for local fluctuations in P, allowing the system to adopt a homogeneous but apolar nematic configuration.” I assume the authors only mean at low lambda 1 as at higher lambda 1 splay and splay bend phases are formed?

I do have some contention with the statement that the splay bend prediction is new. While I agree that in the ferroelectric nematic space where molecules are mostly rodlike this would be a new phase, there is plenty of literature of splay-bend phases and states in dimeric liquid crystals and colloidal systems.

Figure 2b. It strikes me that this texture is highly similar to figure 6 from reference 23. Loverly! The observed textures also clearly show how bend is incorporated at the domain walls. I believe the most commonly invoked domain wall structure is a melting of the polar order at the walls to form a lower density region. Is there any experimental evidence showing that these bend walls are physically reproduced in experiments?

Is figure b and e of the same simulation result? To me it looks like there are some significant differences in the orientation of the polarisation vs director. It would be useful to clarify that these are the same region/result to allow for better comparison.

Is there a particular reason the that order parameter S is so much lower for the Nf phase than for either the splay or splay bend phases? Experimentally I believe S is higher in the Nf phase than in the Ns or N phase.

Have the authors considered why their model didn’t also reproduce the Ntbf phase of Karcz et.al. (10.1126/science.adn6812) and the Nx phase of Zhang et. al. (10.1038/s41467-025-64463-2)?

Reviewer #2

(Remarks to the Author)
In this work, Vats et al develop a compact free energy framework that predicts and explains the structure of all major experimentally observed ferroelectric nematic, splay nematic,
apolar nematic, and isotropic phase. They also predict the possible existence of splay-bend structure. For the splay nematic, does a periodic layer structure exist or not? For the splay-bend structure, what exact angle values between molecular director and polarization direction are suggested for the occurrence of splay-bend structure? Do the authors continuously change the angle as well as the order of magnitude of dipoles and predict their phase behavior?

Reviewer #3

(Remarks to the Author)
In this manuscript, the authors employ a Landau-de Gennes framework, integrating the Q-tensor and polarization vector P, to systematically analyze the bulk phases of ferroelectric nematic liquid crystals (FNLCs). The innovation lies in using dual order parameters to describe the orientational order of the constituent anisotropic molecules. Furthermore, the model predicts a splay-bend phase, which has not yet been experimentally demonstrated.
I have some concerns regarding this manuscript. If the authors can adequately address these issues, I believe the paper is worth publishing.
1. The authors mention that "a generalized description of all observed phases is still lacking" and indicate they have resolved this challenge. However, FNLCs are indeed highly complex systems. For instance, FNLCs possess chiral ground states [Chiral ground states of ferroelectric liquid crystals, Science 383.6689 (2024)]. Furthermore, derivatives of RM734 exhibit an intermediate phase (mesophase or Nx phase), and ion-doped RM734 displays double splay nematic order [Double Splay Nematic Order in Confined Polar Fluids. Phys. Rev. Lett. 134, 238101 (2025)]. There are also other phases such as ferroelectric smectic A, ferroelectric twist-bend, smectic C, etc. Given the complexity of FNLC systems, proposing such a theoretical framework is certainly commendable. Nevertheless, some phrasing in the manuscript should be revised. Expressions like "to capture the full complexity of ferroelectric nematic phases" and "we demonstrate a complete bulk analysis of ferroelectric nematic liquid crystals phases".
2. I think the scenario of decoupled polarization and nematic order has already been investigated in the existing literature [Extended free-energy functionals for achiral and chiral ferroelectric nematic liquid crystals: theory and simulation. Physical Chemistry Chemical Physics 26, 15637-15647 (2024)], and this decoupled model has also been applied in a recent study [Periodically-modulated unipolar and bipolar orders in nematic fluids towards miniaturized nonlinear vectorial optics. Nat. Commun. 16, 9419 (2025)]. The authors need to elaborate on this in the manuscript, specifically addressing how the polarity is decoupled from both the elastic and compressive terms.
3. the current title is somewhat misaligned with the core contribution of the work. Maybe a more appropriate and impactful title would be: "Free Energy Framework for the Bulk Mesophases of Ferroelectric Nematic Liquid Crystals".
4. The bulk free energy f contains numerous coefficients that determine the morphologies of FNLCs. By tuning these values, the authors simulated complex liquid crystalline phases. Regarding the range of these coefficients, could further clarification be provided?
5. In most cases, research on FNLCs is experimentally conducted under confinements. Does the Landau–de Gennes-like free energy framework proposed in this paper have any limitations regarding its applicability under such conditions?
6. Relevant studies have shown that FNLCs possess chiral ground states [Kumari, Priyanka, et al., Chiral ground states of ferroelectric liquid crystals, Science 383.6689 (2024)]. Can this be captured by the proposed bulk ferroelectric nematic liquid crystal model?
7. In the paragraph below Figure 2, it is described that: “…using a perfectly periodic initial condition: the director field was set as (cos θ, sin θ), with θ = θ0 sin (x+y), and the polarization vector was aligned along the director”. Could the authors explain why this particular initial condition was chosen, and whether such an initial alignment occurs naturally during the phase evolution process?
8. Below Equation (1), parameters μ and v are described as "controlling the onset and magnitude of spontaneous polarization". However, later in the text, parameter μ is also referred to as "effectively controls the temperature in our model." I am afraid this may cause confusion.
9. Please supplement the descriptions for the orange and white arrows in Figure 1a. In Figures 1b–d, the black ones could be labeled more clearly. There is an error in the caption of Figure 2: the currently labeled (d) actually refers to Figure 2f.

Version 1:

Reviewer comments:

Reviewer #1

(Remarks to the Author)
The authors have responded to my comments completely. The paper is very suitable for publication in Nature Communications.

Reviewer #3

(Remarks to the Author)
The authors have responded carefully and thoroughly to the majority of my previous comments and suggestions, and the revised manuscript is improved in presentation. upon reviewing the rebuttal and the new manuscript, I still have a few remaining concerns that I believe should be addressed before the work is suitable for publication.

1. The authors argue that depolarization fields are strongly suppressed by screening in the bulk and therefore do not qualitatively alter the bulk phase behavior. While this reasoning is plausible for the uniform ferroelectric nematic phase, it is less convincing for the antiferroelectric splay nematic phase, where the polarization field exhibits pronounced spatial modulations. In such a modulated phase, $\nabla \cdot P \neq 0$ in general, which would give rise to bound charge accumulation and non-negligible depolarization fields even in the bulk. Can the authors provide a more quantitative justification? — for instance, an order-of-magnitude estimate comparing the depolarization energy to the elastic and flexoelectric energy scales at the characteristic modulation wavelength — or to explicitly acknowledge this as a limitation of the current framework.

2. I appreciate that the authors have clarified the theoretical nature of the work in the abstract. Nevertheless, I remain of the view that the current title, Bulk Mesophases of Ferroelectric Nematic Liquid Crystals, reads more like the title of a review article than a theoretical and computational study.

3. The supplementary material currently consists of only 4 pages, containing a brief description of the numerical method and three figures. For a theoretical paper involving a free energy framework with numerous coupled parameters and large-scale numerical simulations, I find this level of supplementary documentation inadequate. I would strongly recommend the authors include the following:

A clear description of the non-dimensionalization scheme, specifying how the model parameters relate to physical (SI) quantities for at least one reference material system;
A systematic convergence test with respect to grid size and time step, demonstrating that the reported phase structures and periodicities are numerically converged;
A table summarizing the physically reasonable ranges of all free energy coefficients, referenced to experimentally accessible quantities;
A derivation, or at least a detailed outline, of the analytical fixed-point solutions presented in Table I.

1. I also note that, as pointed out by Referee 1, the scalar order parameter S is experimentally known to be higher in the ferroelectric nematic phase (N_F) than in the splay nematic phase (N_S), yet the simulation results show the opposite trend. The authors' explanation — that this discrepancy is a consequence of where the two phases happen to sit in the model's parameter space — effectively concedes that the model, in its current parameterization, fails to reproduce the experimentally observed relative ordering of S across the phases.

Version 2:

Reviewer comments:

Reviewer #3

(Remarks to the Author)
The authors have well addressed my concerns. it can be published.

Open Access This Peer Review File is licensed under a Creative Commons Attribution 4.0 International License, which permits use, sharing, adaptation, distribution and reproduction in any medium or format, as long as you give appropriate credit to the original author(s) and the source, provide a link to the Creative Commons license, and indicate if changes were made.
In cases where reviewers are anonymous, credit should be given to 'Anonymous Referee' and the source.
The images or other third party material in this Peer Review File are included in the article's Creative Commons license, unless indicated otherwise in a credit line to the material. If material is not included in the article's Creative Commons license and your intended use is not permitted by statutory regulation or exceeds the permitted use, you will need to obtain permission directly from the copyright holder.
To view a copy of this license, visit https://creativecommons.org/licenses/by/4.0/

Detailed Response to Referees Comments

The detailed responses to comments by the referees are as follows. Page numbers, equation numbers, figure numbers and reference numbers refer to the revised manuscript. The changes have been made in red.

Referee 1: We thank the referee for careful reading of our manuscript, and the constructive comments and suggestions. We have incorporated all of them in the revision, which has led to an improved presentation. Our detailed response is as follows:

C1: The introduction discusses developments to the physics and theory fairly completely. I would say an omission that could be rectified is the inclusion of structure property developments from a chemistry perspective. Particularly in light of their predictive design comments in the abstract. In particularly I believe Phys. Rev. E 104, 014704 (10.1103/PhysRevE.104.014704) should be included as this involved a simple model that has had by fair the biggest impact around predictive development in this space and possibly a few on the development of structure properties relationships in the synthetic space. Of course this is just my opinion and I leave up to the authors.

R1: We thank the the referee and have now expanded the reference list and added following discussion and in para 1 of page 2:

"These systems rely sensitively on molecular design, particularly the presence of asymmetric terminal groups and lateral dipolar components. Even small chemical modifications (e.g., replacing NO_2 with CN) can suppress ferroelectricity despite similar dipole moments, highlighting the role of dipole orientation and intermolecular packing [2, 4, 7]."

We have also added the following line and suggested reference in para 2 of page 7:

"Complementarily, FNLCs have also been modeled using simplified electrostatic interactions of polar rods, capturing the role of density and molecular charge distribution in stabilizing polar order [55]."

C2: This is quite a minor point but the schematic of the Ns phase is not ideal in my opinion. My understanding is that the Ising walls should be vertical i.e. parallel to the director rather here they are cantered to each other leading to a narrowing and widening of the domains.

R2: We thank the referee for pointing this out and have updated the Figure 1.

C3: How do the authors include the alignment of the dipole and molecular long axis. Presumably the director n is coupled to the long axis orientation while the polarization P is couple to the dipole orientation. The authors allude to some angle typically being small (reasonable) but then draw the splay bend phase using a dipole angle of 90 degrees. Could the authors comment more clearly on how they defined this angle in their calculations?

R3: We thank the referee for raising this point. In our formulation, the angle between the director and the polarization is not imposed geometrically. It emerges self-consistently from minimization of the free energy, set by the competition between the coupling terms, and it is then the selection of material parameters which constrains the relative alignment. In a uniform state, the gradient contributions vanish. The anisotropic coupling $\eta\,(\mathbf{P}\cdot\mathbf{Q}\cdot\mathbf{P})$ alone then sets the orientation and favors $\mathbf{P}$ parallel to $\mathbf{n}$. This is the "small angle" regime referred to earlier in the manuscript. In the splay–bend phase, the situation is different. The flexoelectric couplings (λ_1, λ_5, λ_6) dominate the energy balance and drive $\mathbf{P}$ along the gradients of $\mathbf{Q}$. These gradients lie transverse to the local director. This is why the polarization appears perpendicular to the long axis in the figure. We have added the following lines to the manuscript to make this explicit in para 1 of page 9:

"Note that the angle between $\mathbf{P}$ and $\mathbf{n}$ is not imposed geometrically. It emerges self-consistently from minimization of the free energy determined by the competition between flexoelectric and anisotropic coupling terms."

C4: "For $\kappa = 0$, there is no energetic cost for local fluctuations in P, allowing the system to adopt a homogeneous but apolar nematic configuration." I assume the authors only mean at low lambda 1 as at higher lambda 1 splay and splay bend phases are formed?

R4: Thank you. Referee is correct in their assessment. For $\kappa = 0$, there is no energetic cost for local fluctuations in P. However, its coupling to the splay and bend phases can still impact polarization field. To clarify this, we have now modified this line in para 1 of page 9:

"For $\kappa = 0$, there is no energetic cost for local fluctuations in $\mathbf{P}$, allowing the system to adopt a homogeneous but apolar nematic configuration when the flexoelectric couplings are weak."

C5: I do have some contention with the statement that the splay bend prediction is new. While I agree that in the ferroelectric nematic space where molecules are mostly rodlike this would be a new phase, there is plenty of literature of splay-bend phases and states in

dimeric liquid crystals and colloidal systems.

R5: We thank the referee for pointing out this ambiguity. We agree that splay–bend phases have been previously reported in other contexts, such as dimeric liquid crystals and colloidal systems. Here, however, our prediction specifically concerns current ferroelectric nematic liquid crystals, where such a phase has not been established, particularly for predominantly rod-like molecular constituents. We have revised the manuscript to clarify this distinction and have added following lines and references in para 1 of page 9:

"To the best of our knowledge, this phase has not yet been reported experimentally in FNLCs. Though there have been previous reports on analogues phases in other contexts, such as dimeric liquid crystals and colloidal systems [58-64]."

C6: Figure 2b. It strikes me that this texture is highly similar to figure 6 from reference 23. Loverly! The observed textures also clearly show how bend is incorporated at the domain walls. I believe the most commonly invoked domain wall structure is a melting of the polar order at the walls to form a lower density region. Is there any experimental evidence showing that these bend walls are physically reproduced in experiments?

R6: We thank the referee for pointing out the similarity to Fig. 6 of Ref. [23]. Experimentally, the domain walls in the antiferroelectric phase are observed to be of Ising type. The splay-bend walls of the kind that involve director bending have so far been reported in the ferroelectric phase.The bend distortion visible at the walls in Fig. 2b is a feature of the metastable, defect-rich configuration obtained from random initial conditions. The equilibrium antiferroelectric phase has the wall features similar to Fig. 2a and 2c in the experiments. We have added a brief comment in para 1 of page 12 to clarify this point:

"The bend distortion visible at the walls in this metastable state is not characteristic of the equilibrium antiferroelectric phase, where experimentally only Ising-type walls are observed [23]."

C7: Is figure b and e of the same simulation result? To me it looks like there are some significant differences in the orientation of the polarization vs director. It would be useful to clarify that these are the same region/result to allow for better comparison.

R7: Yes, the figure 2(b) and 2(e) represents the director and polarization fields for the same simulation. The noted difference in the orientation of two fields is due to the convergence to a metastable state populated by numerous defects. Although these metastable states exhibits some splay character but these lacks long-range periodicity. We have now explicitly

stated this in para 1 on page 12:

"Sub-figures represent nematic and polarization field for the same set simulation parameters."

C8: Is there a particular reason the that order parameter S is so much lower for the Nf phase than for either the splay or splay bend phases? Experimentally I believe S is higher in the Nf phase than in the Ns or N phase.

R8: We thank the referee for this observation. The apparent lower value of S in N_F phase compared to splay and splay-bend phase in our results is a consequence of where these phases sit in the parameter space of our model, rather than an intrinsic feature of the phases themselves, i.e. they can be changed/adapted/mapped to experiments by using different material coefficients. In our formulation, the flexoelectric couplings λ_i influences S indirectly by setting the equilibrium $\mathbf{P}$, which get coupled to S through anisotropic coupling. Since, the splay and splay-bend phases occur at higher values of λ_i, the resulting S is higher. We agree with the referee's observation and this would be recovered in our formulation if the ferroelectric and modulated phases are compared at similar $|\mathbf{P}|$. We have now added following lines to clarify this in the manuscript in para 1 of page 10:

" We also note that S depends indirectly on the flexoelectric coupling through their effect on equilibrium $|\mathbf{P}|$. Hence phase stabilized at higher λ_i, also exhibit higher S compared to uniform ferroelectric phase."

C9: Have the authors considered why their model didn't also reproduce the Ntbf phase of Karcz et.al. (10.1126/science.adn6812) and the Nx phase of Zhang et. al. (10.1038/s41467-025-64463-2)?

R9: Thank you for the excellent comment. In experiments, the N_{TBF} phase emerges spontaneously as the effective bend elastic coefficient softens due to flexoelectric coupling between the polarization and bend deformation (https://arxiv.org/abs/2602.15687). The resulting bend instability is resolved through the formation of a heliconical structure, since uniform bend cannot fill space. Within our formulation, independent control over the effective splay and bend instabilities is in principle available through the flexoelectric coefficients λ_i, and anisotropic polar susceptibility ($\mu_\parallel$, $\mu_\perp$) is naturally generated by the η coupling. With the additional inclusion of the out-of-plane components of $\mathbf{Q}$, we expect that the N_{TBF} phase should be reproducible within our framework. We agree that this is an interesting direction and can be pursued in future work. The N_X-like two-dimensionally modulated polar

mesophase may arise from our framework, consisting of a periodic lattice of splay domains with alternating polarity. However, a detailed quantitative comparison would be required to support such an identification, and can be taken up in future studies. To suggest these possibilities, we have now added following lines and additional references in our revised manuscript in para 1 of page 18:

"The phenomenology of FNLCs is very rich with phases reported in the literature-such as the chiral ferroelectric nematic, the twist–bend ferroelectric (N_{TBF}), the N_X mesophase, and ferroelectric smectic phases [12, 19, 24-28, 53]. In addition, the framework can be naturally extended to confined FNLC systems by including surface anchoring and surface-originating electrostatic contributions. These represent natural directions of future extension of the work."

Referee 2:

C1: In this work, Vats et al develop a compact free energy framework that predicts and explains the structure of all major experimentally observed ferroelectric nematic, splay nematic, apolar nematic, and isotropic phase. They also predict the possible existence of splay-bend structure. For the splay nematic, does a periodic layer structure exist or not? For the splay-bend structure, what exact angle values between molecular director and polarization direction are suggested for the occurrence of splay-bend structure? Do the authors continuously change the angle as well as the order of magnitude of dipoles and predict their phase behavior?

R1: We thank the referee for the thoughtful questions. We address each in turn.

The splay nematic phase in our calculations exhibits a periodic modulation. The director and polarization fields vary spatially with a well-defined periodicity determined by the competition between the flexoelectric coupling and the elastic terms. The resulting structure has alternating splay domains forming a layer-like periodic pattern, consistent with the splay nematic phase reported in Ref. [23]. This periodicity emerges from the minimization and is not imposed explicitly. We have shown the periodicity of the splay structures for different λ_1 in the supplementary figure 1. We have added the following lines to clarify this point in para 1 of page 10:

"The splay nematic phase exhibits a periodic, layer-like modulation of the director and polarization fields. The periodicity is not imposed apriori but emerges from the free-energy minimization through the balance between the flexoelectric couplings and the elastic terms."

Similarly as in response to Referee 1, comment 3, the angle between **n** and **P** is not a prescribed input to our model. It emerges self-consistently from the competition between the different coupling terms. In the ferroelectric nematic phase, the anisotropic coupling dominates and the angle is close to zero. In the splay–bend phase, the local angle is close to 90°, since the relevant gradient of **Q** is transverse to the local director. There is therefore no single exact value of the angle that characterizes the splay–bend phase, but the dominant orientation in the parameter regime we explored is close to perpendicular (i.e. relative angle variation below 15°). We have also added this discussion to the manuscript in paragraph 1 of page 9:

"Note that the angle between **P** and **n** is not imposed geometrically. It emerges self-consistently from minimization of the free energy determined by the competition between flexoelectric and anisotropic coupling terms."

Furthermore, we have explored the phase behavior as a function of λ_i, η, and the polar coefficients μ and ν, which together set both the angle and the magnitude of $|\mathbf{P}|$. Varying these parameters produces a continuous evolution of the system through the different phases, as shown in the phase diagram. This parameter scan is therefore equivalent to a continuous variation of the magnitude of **P**.

Referee 3:

C1: The authors mention that "a generalized description of all observed phases is still lacking" and indicate they have resolved this challenge. However, FNLCs are indeed highly complex systems. For instance, FNLCs possess chiral ground states [Chiral ground states of ferroelectric liquid crystals, Science 383.6689 (2024)]. Furthermore, derivatives of RM734 exhibit an intermediate phase (mesophase or Nx phase), and ion-doped RM734 displays double splay nematic order [Double Splay Nematic Order in Confined Polar Fluids. Phys. Rev. Lett. 134, 238101 (2025)]. There are also other phases such as ferroelectric smectic A,

ferroelectric twist-bend, smectic C, etc. Given the complexity of FNLC systems, proposing such a theoretical framework is certainly commendable. Nevertheless, some phrasing in the manuscript should be revised. Expressions like "to capture the full complexity of ferroelectric nematic phases" and "we demonstrate a complete bulk analysis of ferroelectric nematic liquid crystals phases".

R1: Thank you. Fully, as suggested, we have now corrected these statements at different parts in the manuscript.

In para 2 of page 3: "Landau–de Gennes-like free energy framework is developed that incorporates flexoelectric interactions, polarization gradient elasticity, and anisotropic coupling within a unified theoretical framework, enabling systematic exploration of both static and dynamic FNLC phenomena."

In para 1 of page 17: "In this work, we demonstrate bulk analysis of ferroelectric nematic liquid crystal phases, providing mesoscopic insight into the structure of observed nematic ferroelectric phases-ferroelectric nematic, antiferroelectric splay nematic, apolar nematic, and isotropic phase- and also predict a possible new splay-bend phase, if the orientational bond between the nematic director and polarization could be energetically reduced."

We have also added following lines in para 1 of page 18 to explicitly discuss the mentioned phases mentioned and we have extended the reference list:

"The phenomenology of FNLCs is very rich with phases reported in the literature-such as the chiral ferroelectric nematic, the twist–bend ferroelectric (N_{TBF}), the N_X mesophase, and ferroelectric smectic phases [12, 19, 24-28, 53]. In addition, the framework can be naturally extended to confined FNLC systems by including surface anchoring and surface-originating electrostatic contributions. These represent natural directions of future extension of the work."

C2: I think the scenario of decoupled polarization and nematic order has already been investigated in the existing literature [Extended free-energy functionals for achiral and chiral ferroelectric nematic liquid crystals: theory and simulation. Physical Chemistry Chemical Physics 26, 15637-15647 (2024)], and this decoupled model has also been applied in a recent study [Periodically-modulated unipolar and bipolar orders in nematic fluids towards miniaturized nonlinear vectorial optics. Nat. Commun. 16, 9419 (2025)]. The authors need to elaborate on this in the manuscript, specifically addressing how the polarity is decoupled

from both the elastic and compressive terms.

R2: We thank the referee. As suggested we now fully elaborate the mentioned approach and also we have included the suggested references. Specfically, we have included the following discussion to address their point in para 2 of page 7:

"In our framework, the nematic and polar orders are treated as independent fields, with bulk elastic terms decoupled from each other. The polar field is coupled to the elasticity through the flexoelectric couplings. Similar treatment of the nematic and polar order parameters as independent variables has been employed in recent studies [54, 56, 57], where the authors construct free energy for different mesophases. The compressive term, relevant to chiral polar helices [53], is in our case not included as the present work concerns achiral bulk phases."

C3: the current title is somewhat misaligned with the core contribution of the work. Maybe a more appropriate and impactful title would be: "Free Energy Framework for the Bulk Mesophases of Ferroelectric Nematic Liquid Crystals".

R3: We thank the referee for the suggestion. To address the underlying concern that the theoretical nature of the work should be clear to the reader, we have revised the abstract to state more explicitly that we develop a Landau-de Gennes-like free energy framework for these phases.

C4: The bulk free energy f contains numerous coefficients that determine the morphologies of FNLCs. By tuning these values, the authors simulated complex liquid crystalline phases. Regarding the range of these coefficients, could further clarification be provided?

R4: We thank the referee for the comment. To clarify how the coefficients in f are constrained, we have added the following paragraph on page 6 of the revised manuscript, outlining how each coefficient can in principle be linked to measurable experimental quantities and thereby to physically reasonable ranges:

"The free energy in Eq. (1) has multiple coefficients, which can be linked to different experimentally measured quantities. For example, A, B, C may be determined from the birefringence, the isotropic–nematic transition temperature, and the latent heat from DSC [50]. Elastic constant L is related to the Frank constants, accessible through Frederiks-threshold measurements [50, 52]. μ and ν can be inferred from the spontaneous polarization $\mathbf{P}(T)$. Similarly, the polar stiffness κ could be determined from the width of polar domain

walls in SHG microscopy [2, 8, 22]. The anisotropic coupling η can be accessed through the equilibrium angle between P and the director, accessible by SHG interferometry. The flexoelectric couplings coefficients λ_i are constrained through their effect on the splay and bend elastic constants. For example, integrating out **P** in the paraelectric regime suggests effective constants $K_1^{eff} = 2LS^2 - e_1^2/\mu_{\parallel}$ and $K_3^{eff} = 2LS^2 - e_3^2/\mu_{\perp}$ in our formulation. Here e_1 and e_3 are linear combinations of the λ_i (see Eq. (4)) and $\mu_{\parallel} = \mu - 2\eta S\left(1 - \frac{1}{d}\right)$ and $\mu_{\perp} = \mu + \frac{2\eta S}{d}$. These effective elastic constants are measured directly in the experiments [9, 22, 53]. We believe such a joint set of measurements -similar to those in other non-ferroelectric liquid crystal materials- can provide good constraints on the different FNLC material parameter values."

C5: In most cases, research on FNLCs is experimentally conducted under confinements. Does the Landau–de Gennes-like free energy framework proposed in this paper have any limitations regarding its applicability under such conditions?

R5: We thank the referee for this important question. The free energy framework presented in this work is formulated specifically for the bulk, to avoid possible effects from the confining surfaces. However, the approach and the free energy does not have any fundamental limitation that would prevent its application to systems under confinement. Indeed, Landau-de Gennes models are highly capable for studying confined systems, as the free energy density can be easily extended with surface free energy terms. Specifically, under confinement, the current bulk free energy will require additional surface anchoring terms and/or surface originating electrostatic contributions while the bulk terms will stay unchanged. To emphasise this, we have now added a remark on this in the manuscript in para 1 of page 18:

"In addition, the framework can be naturally extended to confined FNLCs by including surface anchoring and surface-originating electrostatic contributions. These represent natural directions of future extension of the work."

C6: Relevant studies have shown that FNLCs possess chiral ground states [Kumari, Priyanka, et al., Chiral ground states of ferroelectric liquid crystals, Science 383.6689 (2024)]. Can this be captured by the proposed bulk ferroelectric nematic liquid crystal model?

R6: Our present work focuses on the bulk free energy. The framework can be naturally extended to include electrostatic (depolarization) contributions and surface energy. Such an extension, minimized in a slab geometry, would provide the necessary ingredients to investi-

gate the chiral ground state reported in that work. We note that simply adding a chiral term to the free energy would instead produce a helix of fixed handedness, which is qualitatively distinct from the coexisting left- and right-handed domains observed experimentally. We have revised the manuscript to include this point in para 1 of page 18:

"The phenomenology of FNLCs is very rich with phases reported in the literature-such as the chiral ferroelectric nematic, the twist–bend ferroelectric (N_{TBF}), the N_X mesophase, and ferroelectric smectic phases [12, 19, 24-28, 53]. In addition, the framework can be naturally extended to confined FNLC systems by including surface anchoring and surface-originating electrostatic contributions. These represent natural directions of future extension of the work."

C7: In the paragraph below Figure 2, it is described that: "…using a perfectly periodic initial condition: the director field was set as (cos θ, sinθ), with $\theta = \theta$ sin (x+y), and the polarization vector was aligned along the director". Could the authors explain why this particular initial condition was chosen, and whether such an initial alignment occurs naturally during the phase evolution process?

R7: We thank the referee for this question. The periodic initial conditions are chosen as a numerical convenience to seed the simulation with a spatial modulation of the director. The seed does not impose a fixed periodicity and only provide a weak orientational modulation. The system selects its own periodicity through the competition between different coupling terms. This choice is motivated by a purely practical consideration. As shown in Fig. 2(b) and (e), simulations initialized from a isotropic or random state get trapped in metastable configurations with numerous defects. Seeding the simulation with a modulated director avoids this issue and allows a systematic comparison of the equilibrium structures across the parameter space. We have now rephrased the following sentences for clarity in para 1 of page 12 :

"This seeded configuration introduces a spatially modulated orientation without imposing a fixed wavelength, allowing the system to spontaneously select its periodicity through competition between different coupling terms. This choice is motivated in order to avoid formation of metastable states."

C8: Below Equation (1), parameters μ and ν are described as "controlling the onset and magnitude of spontaneous polarization". However, later in the text, parameter μ is also referred to as "effectively controls the temperature in our model." I am afraid this may

cause confusion.

R8: We thank the referee for pointing this out. We have now modified the following for providing more clarity in para 2 of page 5:

"The parameter with $\mu = \mu_0(T - T_C)$ effectively controls the temperature. T is the quench temperature and T_C is the effective transition temperature for the polar-to-apolar transition. μ_0 and ν control the onset and magnitude of the spontaneous polarization. κ describes spatial polarization stiffness and penalizes the local variation in the polarization field."

C9: Please supplement the descriptions for the orange and white arrows in Figure 1a. In Figures 1b–d, the black ones could be labeled more clearly. There is an error in the caption of Figure 2: the currently labeled (d) actually refers to Figure 2f.

R9: We thank the referee for flagging the caption error, which has now been corrected. As suggested, the labeling on Fig 1 was improved and text is added into the caption to Fig 1:

"The orange (white) arrow represents the direction of $\mathbf{P}$ pointing in the up (down) direction."

Detailed Response to Referees Comments

The detailed responses to comments by the referees are as follows. Page numbers, equation numbers, figure numbers and reference numbers refer to the revised manuscript. The changes have been made in red.

Referee 1:

We thank the referee for accepting the manuscript for publication.

Referee 3: We thank the referee for the constructive comments and suggestions. We have incorporated all of them in the revision, which has led to an improved presentation. Our detailed response is as follows:

C1: The authors argue that depolarization fields are strongly suppressed by screening in the bulk and therefore do not qualitatively alter the bulk phase behavior. While this reasoning is plausible for the uniform ferroelectric nematic phase, it is less convincing for the antiferroelectric splay nematic phase, where the polarization field exhibits pronounced spatial modulations. In such a modulated phase, $\nabla \cdot P \neq 0$ in general, which would give rise to bound charge accumulation and non-negligible depolarization fields even in the bulk. Can the authors provide a more quantitative justification? — for instance, an order-of-magnitude estimate comparing the depolarization energy to the elastic and flexoelectric energy scales at the characteristic modulation wavelength — or to explicitly acknowledge this as a limitation of the current framework.

R1: We thank the referee for the observation. In the modulated phase, $\nabla \cdot \mathbf{P}$ gives rise to bound charges and hence the depolarization field in the bulk. We agree with the referee that in the modulated phase, $\nabla \cdot P \neq 0$ which would give rise to the bound charge accumulation and non-negligible depolarization field. In experiments, this effect is partially regularized due to the screening by free ions and by the geometrically alternating sign of the bound charges. The net effect of the depolarization field is the increase in the effective splay constant [https://doi.org/10.1103/PhysRevE.111.L053402], which modifies the location of phase boundaries and corresponding periodicity values. It has been established that the antiferroelectric splay nematic phase occurs via softening of the splay coefficient resulting

from the flexoelectric coupling. The flexoelectrically-driven onset of the splay nematic phase is therefore captured by our framework, while the depolarization field — which we have not included — would modify the equilibrium modulation period and the location of the phase boundaries. As suggested by the referee, we have now added following paragraph in page 8: "One limitation of the work is that we have not included the effects of the depolarization fields [15, 18, 49] explicitly. In the uniform bulk phase, the effects of the depolarization field are strongly suppressed by screening, and their primary influence manifests near interfaces or in confined geometries [9, 23]. In the modulated phase, $\nabla \cdot \mathbf{P} \neq 0$ generates bound charges and a corresponding depolarization field, which increases the effective splay constant [49]. Its inclusion would shift the equilibrium modulation period and the location of the phase boundaries, and is a natural direction for a more quantitatively accurate description."

C2: I appreciate that the authors have clarified the theoretical nature of the work in the abstract. Nevertheless, I remain of the view that the current title, Bulk Mesophases of Ferroelectric Nematic Liquid Crystals, reads more like the title of a review article than a theoretical and computational study.

R2: As suggested, we have now changed the title of the manuscript to "Bulk mesophases modeling of ferroelectric nematic liquid crystals".

C3: The supplementary material currently consists of only 4 pages, containing a brief description of the numerical method and three figures. For a theoretical paper involving a free energy framework with numerous coupled parameters and large-scale numerical simulations, I find this level of supplementary documentation inadequate. I would strongly recommend the authors include the following:

1. A clear description of the non-dimensionalization scheme, specifying how the model parameters relate to physical (SI) quantities for at least one reference material system;
2. A systematic convergence test with respect to grid size and time step, demonstrating that the reported phase structures and periodicities are numerically converged;
3. A table summarizing the physically reasonable ranges of all free energy coefficients, referenced to experimentally accessible quantities;
4. A derivation, or at least a detailed outline, of the analytical fixed-point solutions presented in Table I.

R3: As suggested by the reviewer, we have now extensively expanded the entire supplementary information in accordance with the suggested points above.

C4: I also note that, as pointed out by Referee 1, the scalar order parameter S is experimentally known to be higher in the ferroelectric nematic phase (N_F) than in the splay nematic phase (N_S), yet the simulation results show the opposite trend. The authors' explanation — that this discrepancy is a consequence of where the two phases happen to sit in the model's parameter space — effectively concedes that the model, in its current parameterization, fails to reproduce the experimentally observed relative ordering of S across the phases.

R4: We thank the referee for this important observation. The figures in the main text show the different phases at different values of the flexoelectric coupling λ_i. Different choices of λ_i corresponds to different ferroelectric nematic materials. Hence, in this comparison the relative ordering of S did not follow the experimental trend, as correctly noted by the referee. We have examined the behavior of S along a single trajectory in the μ, with all other coefficients held fixed. We note that the system goes through ferroelectric (N_F) $\rightarrow$ antiferroelectric splay nematic (N_S) $\rightarrow$ nematic (N) phases as μ increases. Along this path, the average scalar order parameter decreases monotonically, with its largest value in the ferroelectric phase. We have now depicted this in the supplementary material Fig. S4. We have also added the following lines in the main text in para 1 of page 19:

Along this trajectory, S increases from the nematic to the splay-nematic to the ferroelectric phase, reflecting the mutual coupling of polar and orientational order in the free energy. We present this as a qualitative feature of the model rather than a quantitative comparison to a specific material.

Also, in para 1 of page 9:

Since these coefficients encode material-specific properties, different choices of λ_i and κ correspond to different ferroelectric nematic materials, and the phase diagrams thus map how the bulk behavior varies across the material family.

Detailed Response to Referees Comments

Referee 3:

We thank the referee for accepting the manuscript for publication. Their comments and suggestions has led to an improved presentations.
